# Jag1 represses Notch activation in lateral supporting cells and inhibits an outer hair cell fate in the medial cochlea

**DOI:** 10.1242/dev.202949

**Published:** 2024-11-05

**Authors:** Sandra de Haan, Agustin A. Corbat, Christopher R. Cederroth, Lisa G. Autrum, Simona Hankeova, Elizabeth C. Driver, Barbara Canlon, Matthew W. Kelley, Emma R. Andersson

**Affiliations:** ^1^ Department of Cell and Molecular Biology, Karolinska Institutet, Stockholm 17177, Sweden; ^2^ Laboratory of Cochlear Development, National Institute on Deafness and Other Communication Disorders, National Institutes of Health, Bethesda, MD 20892, USA; ^3^ Department of Physiology and Pharmacology, Karolinska Institutet, Stockholm 17177, Sweden; ^4^ Translational Hearing Research, Tübingen Hearing Research Center, Department of Otolaryngology, Head and Neck Surgery, University of Tübingen, Tübingen 72074, Germany; ^5^ Department of Structural Biology, Genentech, South San Francisco, CA 94080, USA

**Keywords:** Jag1, Outer hair cell-like cell, Notch, Cochlea

## Abstract

Notch signaling patterns the cochlear organ of Corti, and individuals with the *JAG1*/*NOTCH2*-related genetic disorder Alagille syndrome can thus experience hearing loss. We investigated the function of Jag1 in cochlear patterning and signaling using *Jag1^Ndr/Ndr^* mice, which are a model of Alagille syndrome. *Jag1^Ndr/Ndr^* mice exhibited expected vestibular and auditory deficits, a dose-dependent increase in ectopic inner hair cells, and a reduction in outer hair cells. Single cell RNA sequencing of the organ of Corti demonstrated a global dysregulation of genes associated with inner ear development and deafness. Analysis of individual cell types further revealed that Jag1 represses Notch activation in lateral supporting cells and demonstrated a function for Jag1 in gene regulation and development of outer hair cells. Surprisingly, ectopic ‘outer hair cell-like’ cells were present in the medial compartment and pillar cell region of *Jag1^Ndr/Ndr^* cochleae, yet they exhibited location-dependent expression of the inner hair cell fate-determinant *Tbx2*, suggesting Jag1 is required for *Tbx2* to drive inner hair cell commitment. This study thus identifies new roles for Jag1 in supporting cells, and in outer hair cell specification and positioning.

## Teaser

**Summary:** Jag1-regulated Notch signaling controls cochlear patterning, affecting inner and outer hair cell specification, and hearing in Alagille syndrome, as shown by the *Jag1^Ndr/Ndr^* mouse model.

## INTRODUCTION

Proper auditory function requires a precisely ordered mosaic pattern of mechanosensitive inner hair cells (IHCs) and outer hair cells (OHCs), as well as several subtypes of associated supporting cells (SCs) within the organ of Corti. Organ of Corti formation is controlled by a series of cell fate decisions mediated by Notch signaling, a conserved signaling pathway acting through membrane-bound ligand–receptor interactions. Two distinct Notch-mediated signaling mechanisms drive pattern formation: lateral induction, by which the Notch ligand JAG1 activates Notch and forms the prosensory domain containing progenitor cells that will give rise to the variety of cell types in the organ of Corti; and lateral inhibition, by which the Notch ligands DLL1 and JAG2 repress the hair cell (HC) fate in contacting cells. Both signaling modes rely on trans-signaling events, in which Notch receptors are activated by Notch ligands on juxtaposed cells.

In humans, *JAG1* mutations are associated with the congenital disorder Alagille syndrome (ALGS) (Alagille et al., 1975; Li et al., 1997; Oda et al., 1997). Although ALGS is best known as a liver disorder, patients also exhibit hearing deficits including conductive and sensorineural hearing loss, which are due to middle ear bone malformations or sensory and/or neural cell deficits, respectively (Teng et al., 2017). Balance can also be affected in patients, as temporal bone defects, including loss of the posterior semicircular canal of the vestibular system, have been reported (Okuno et al., 1990). Finally, individuals with *JAG1* mutations and hearing deficits but no liver involvement have also been reported (Le Caignec et al., 2002). Understanding how *Jag1* insufficiency impacts ear development in a model of ALGS could thus provide deeper insights into hearing loss in patients.

The best-described Notch loss-of-function phenotype, induced by *Notch1* deletion or by a gamma-secretase inhibitors, is an excess of IHCs and OHCs at the expense of SCs, which arises when the HC fate is not repressed by Notch activation in SCs (Brown and Groves, 2020) (for an overview of Notch defective models, see Brown and Groves, 2020, and Table S1). In contrast to Jag2 and Dll1, which are expressed only in developing HCs, Jag1 is expressed during the entire time window of cochlear development: from early formation of the prosensory domain, then alongside *Jag2* and *Dll1* during cell fate acquisition, and finally during cochlear maturation, when its expression is maintained in SCs. Defects in Jag1 result in supernumerary IHCs but fewer OHCs, and subsequent hearing deficits, as exemplified by mice with mutations in *Jag1* (Chrysostomou et al., 2020; Gilels et al., 2022; Kiernan et al., 2006; Vrijens et al., 2006) (Tables S1, S2). More recently, conditional deletion of Jag1 in SCs was shown to result in the absence of Hensen's cells (HeCs) (Chrysostomou et al., 2020; Morrison et al., 1999). Previous reported models, however, are limited to conditional deletion and heterozygous loss of function of *Jag1* due to late embryonic lethality of *Jag1* germline mutants. It therefore remains elusive how *Jag1* insufficiency affects the development of individual SCs and HCs, in particular IHCs and OHCs, both at the transcriptomic level and in cellular patterning.

Here, we investigate the role of Jag1-mediated Notch signaling in cochlear patterning and signaling, using the ‘Nodder’ (*Jag1^Ndr/Ndr^*) mouse model for ALGS, which is, in contrast to previously reported models, viable in the homozygous condition. The JAG1^NDR^ missense mutant is expressed *in vivo*, and traffics normally (Hansson et al., 2010), and does not bind or activate NOTCH1, but can partially activate NOTCH2, resulting in Jag1 insufficiency (Andersson et al., 2018; Hansson et al., 2010). *Jag1^+/Ndr^* and *Jag1^Ndr/Ndr^* mice exhibited severe patterning defects, including a dose-dependent increase in ectopic IHCs, and a reduction in OHCs, with a concomitant increase and decrease in associated medial and lateral SCs, respectively. Transcriptomic analyses of *Jag1^Ndr/Ndr^* cochleae demonstrated a global dysregulation of genes associated with inner ear development and deafness. Single cell RNA sequencing (scRNAseq) furthermore revealed an upregulation of Notch target genes in *Jag1^Ndr/Ndr^* lateral SC populations, indicating that Jag1 limits Notch signaling in lateral SCs. In addition, there was major transcriptomic dysregulation in OHCs, revealing a function for *Jag1* in OHC gene regulation and development. Finally, we identified ectopic SLC26A5 (Prestin) positive ‘outer hair cell-like’ (OHC-like) cells in the IHC and pillar cell (PC) compartments, with OHC-like cells in the IHC compartment expressing the IHC fate regulator *Tbx2.* These findings indicate that *Tbx2* is not sufficient to drive IHC commitment in OHC-like cells in the *Jag1*-mutant IHC compartment, and highlights a previously unreported role for Notch signaling in IHC versus OHC fate acquisition.

## RESULTS

### Adult *Jag1^Ndr/Ndr^* mice exhibit major vestibular and auditory defects

**Fig. 1.**
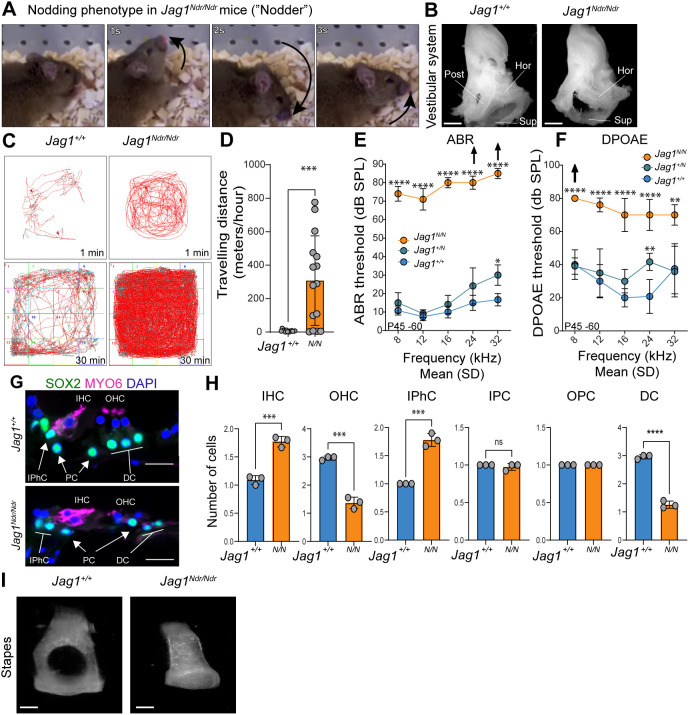
**Adult *Jag1^Ndr/Ndr^* mice exhibit major vestibular and auditory defects.** (A) Timelapse images of head nodding behavior of *Jag1^Ndr/Ndr^* mice, showing movement of the head upwards and towards the shoulders. (B) *Jag1^+/Ndr^* and *Jag1^Ndr/Ndr^* vestibular systems, showing the absence of the posterior semicircular canal in *Jag1^Ndr/Ndr^* mice*.* (C,D) Open field test tracking for 1 or 30 min, demonstrating that (C) *Jag1^Ndr/Ndr^* mice run in circles and (D) are hyperactive, as measured by meters travelled per hour (distance travelled=307.7±267.8 m/h in *Jag1^Ndr/Ndr^* versus 7.5±5.8 m/h in *Jag1*^+/+^; *P*<0.001, data are mean±s.d.)*.* (E,F) Auditory measurements indicate hearing loss with increased ABR thresholds (E) and DPOAE thresholds (F) in *Jag1^Ndr/Ndr^* mice. Arrows indicate no auditory response was measured at the highest sound stimulus. (G) HC and SC phenotype at adult stage (P50) indicates a disorganized pattern of *Jag1^Ndr/Ndr^* HCs (MYO6^+^ cells, red) and SCs (Sox2^+^ cells, green). (H) Quantification of HC and SC phenotype at P50. (I) *Jag1^Ndr/Nd^* display malformed columnar stapes*.* Data are mean±s.d.; each datapoint in D represents measurements from one mouse. Scale bars: 20 µm. **P<0.01; ***P<0.001, ****P<0.0001 [unpaired *t*-test (D,H); one-way ANOVA with Bonferroni correction for auditory measurements, *n*=6 per genotype (E,F)]. Post, posterior; Hor, horizontal; Sup, superior; ABR, auditory brain stem response; DPOAE, distortion product otoacoustic emission; DC, Deiter’s cell; HC, hair cell; SC, supporting cell; IHC, inner hair cell; IPhC, inner phalangeal cell; OHC, outer hair cell; OPC, outer pillar cell.

To assess the impact of the *Jag1* Nodder mutation on functional characteristics of the inner ear, we first assessed balance and hearing function in *Jag1^Ndr/Ndr^* mice (‘Nodder’ mice), which recapitulate major hallmarks of ALGS (Andersson et al., 2018). As reported previously, but not shown, *Jag1^Ndr/Ndr^* mice exhibit a repetitive up-down head movement, with emphasis on head tossing upwards towards the shoulders (Fig. 1A, 1 s panel; Movie 1). Analysis of the vestibular system at postnatal day (P)45-P60 demonstrated an absence of the posterior semicircular canal, which detects head-tilting towards the shoulders (Fig. 1B). In addition to the distinctive head-nodding behavior, *Jag1^Ndr/Ndr^* exhibit circling behavior and are hyperactive (Fig. 1C,D).

Hearing loss is reported in ALGS (Le Caignec et al., 2002; Saleh et al., 2016; Spinner et al., 1993; Teng et al., 2017), and in heterozygous mutant and *Jag1* conditional loss-of-function mice (Chrysostomou et al., 2020; Gilels et al., 2022; Kiernan et al., 2006; Vrijens et al., 2006) (Table S2). We therefore assessed hearing function by measuring auditory brain stem response (ABR) and distortion product otoacoustic emissions (DPOAE) in adult (P45-P60) *Jag1^+/+^*, *Jag1^+/Ndr^* and *Jag1^Ndr/Ndr^* mice. *Jag1^Ndr/Ndr^* mice had severe hearing deficits with ABR thresholds above 70 decibels (dB SPL) along all measured frequencies, whereas *Jag1^+/Ndr^* mice had a mild hearing deficit only at higher frequencies (Fig. 1E). Additionally, DPOAE thresholds were elevated among all frequencies in *Jag1^Ndr/Ndr^* mice, indicating reduced cochlear amplification and OHC dysfunction (Fig. 1F). Histological examination of the adult cochlea demonstrated an increase in the number of IHCs and IPhCs, and a decrease in OHC and DCs, indicating patterning defects that could result in sensorineural hearing loss, as seen in patients with ALGS (Teng et al., 2017) (Fig. 1G,H). As neuronal defects could also contribute to sensorineural hearing loss, we further studied ABR characteristics and observed delayed ABR wave 1 responses, in the absence of changes in spiral ganglion nuclei abundance (Fig. S1), indicating that hearing loss might be due to both patterning defects and reduced auditory nerve conduction. Finally, as both sensorineural and conductive hearing loss are reported in Alagille syndrome, we assessed the middle ear bones to determine whether conductive function is compromised and found that *Jag1^Ndr/Ndr^* mice exhibited malformed stapes (Fig. 1I). In summary, *Jag1^Ndr/Ndr^* mice exhibit profound balance and hearing defects, with vestibular and middle ear bone malformations, and organ of Corti patterning defects, which together recapitulate the mixed hearing loss in individuals with Alagille syndrome.

### Inner ear development and hearing genes are dysregulated in the *Jag1^Ndr/Ndr^* organ of Corti

**Fig. 2.**
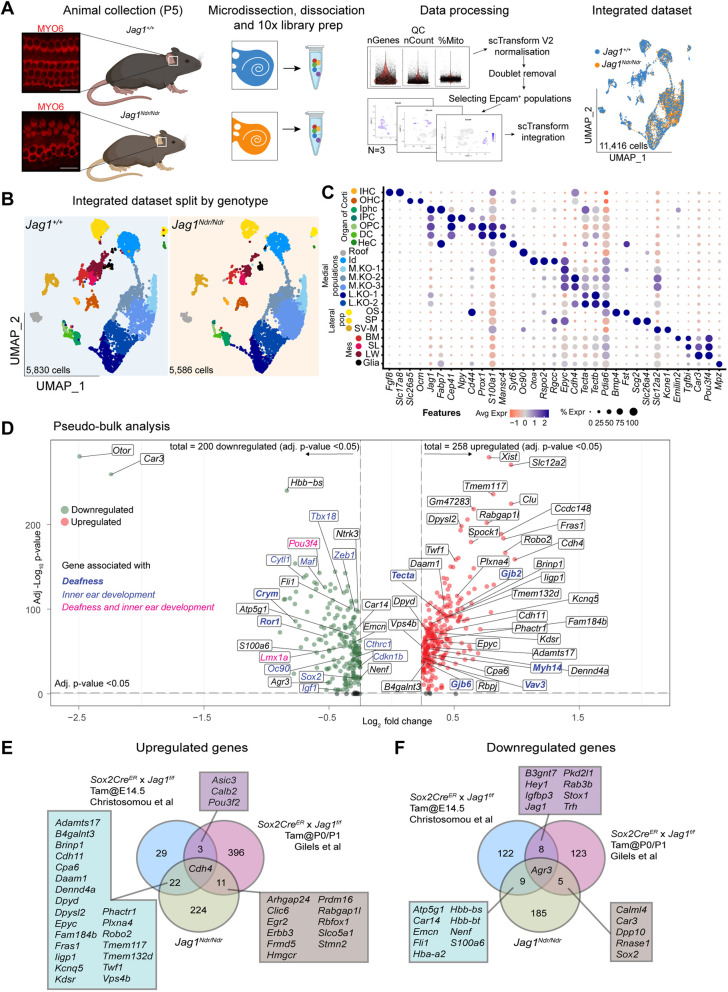
**Inner ear development and hearing genes are dysregulated in the *Jag1^Ndr/Ndr^* organ of Corti.** (A) Summary image of scRNAseq workflow. Scale bars: 20 µm. (B) UMAP of *Jag1^Ndr/Ndr^* and *Jag^+/+^* integrated datasets (*n*=3 per genotype), split by genotype. (C) Dot plot showing marker genes used to identify and annotate different cell populations. (D) Volcano plot showing pseudo-bulk downregulated genes (left, green) and upregulated genes (right, red) between *Jag1^Ndr/Ndr^* and *Jag^+/+^*, highlighting genes associated with deafness (blue, bold), inner ear development (blue) or both (magenta). (E,F) Venn diagrams showing the overlap between pseudo-bulk analysis identified (E) upregulated and (F) downregulated genes in the *Jag1^Ndr/Ndr^* dataset compared with genes previously reported to be up- or downregulated in Jag1 defective models. DC, Deiter's cell; HC, hair cell; SC, supporting cell; IHC, inner hair cell; IPhC, inner phalangeal cell; OHC, outer hair cell; OPC, outer pillar cell; HeC, Hensen's cell; Id, interdental; OS, outer sulcus; M.KO, medial Köllikers organ; L.KO, lateral Kölliker's organ; SP, spiral prominence; SV-M, stria vascularis marginal; BM, basement membrane; SL, spiral limbus; LW, lateral wall.

*Jag1* loss of function results in supernumerary IHCs, a reduction in OHCs (Kiernan et al., 2001; Tsai et al., 2001; Vrijens et al., 2006) and SC survival defects (Campbell et al., 2016; Chrysostomou et al., 2020; Gilels et al., 2022), and disorganized HCs and SCs were present in both adult *Jag1^Ndr/Ndr^* mice (Fig. 1G) and at postnatal day P5 (Fig. 2A). Therefore, to investigate the consequences of JAG1 insufficiency in individual cell types in *Jag1^Ndr/Ndr^* mice, we characterized transcriptional changes in the *Jag1^Ndr/Ndr^* organ of Corti by scRNAseq. We collected and sequenced cells from the organ of Corti of *Jag1^+/+^* and *Jag1^Ndr/Ndr^* mice at P5, when individual cell types of the organ of Corti have differentiated and are undergoing maturation (Walters and Zuo, 2013). After quality control and filtering, 11,416 cells were analyzed (Fig. 2A,B). Dimensionality reduction using UMAP and clustering identified 21 cell types/states, including different cell types within, and medial and lateral to the organ of Corti, as well as periotic mesenchyme and glial populations (Fig. 2B,C, Table S3).

To compare the scRNAseq results with previously published *Jag1*-related bulk RNAseq datasets (Chrysostomou et al., 2020; Gilels et al., 2022), we first performed a pseudo-bulk analysis of the *Jag1^Ndr/Ndr^* and *Jag1^+/+^* populations. Differential gene expression analysis for the cochlear epithelium dataset revealed 458 dysregulated genes (avg. Log 2 FC >0.25, or <−0.25, and adj. *P*-value<0.05), including genes linked to deafness (https://www.ebi.ac.uk/gwas/efotraits/EFO_0004238, Fig. 2D, Table S4, such as *Pou3f4* (Bitner-glindzicz et al., 1995; De Kok et al., 1995), *Crym* (Abe et al., 2003), *Ror1* (Diaz-Horta et al., 2016) and *Lmx1a* (Schrauwen et al., 2018), and contributing to the GO terms ‘Inner ear development’ and ‘Inner ear morphogenesis’ (Fig. 2D, Table S4, GO:0048839 and GO:0042471). *Jag1^Ndr/Ndr^* dysregulated genes overlapped with genes dysregulated upon *Jag1* silencing in SCs (Chrysostomou et al., 2020; Gilels et al., 2022), specifically upregulation of the medial SC marker *Cdh4* (Burns et al., 2015) in all models, and downregulation of *Agr3*, which is most strongly expressed in lateral SCs (Jean et al., 2023) (Fig. 2E,F). Therefore, the *Jag1^Ndr/Ndr^* inner ear exhibits a dysregulated gene signature associated with inner ear development, morphogenesis and hearing, and recapitulates aspects of published *Jag1* loss-of-function transcriptomic data.

### *Jag1^Ndr/Ndr^* mice exhibit a medial boundary defect, a reduction in lateral SCs, and HeC ablation

As the pseudo-bulk analysis demonstrated an overlap in dysregulated genes between *Jag1^Ndr/Ndr^* mice and models with conditional deletion of *Jag1* in SOX2-expressing SCs (Fig. 2E,F), we next assessed SC patterning and abundance, as well as transcriptional changes for each of the SC subtypes in *Jag1^Ndr/Ndr^* mice.

**Fig. 3.**
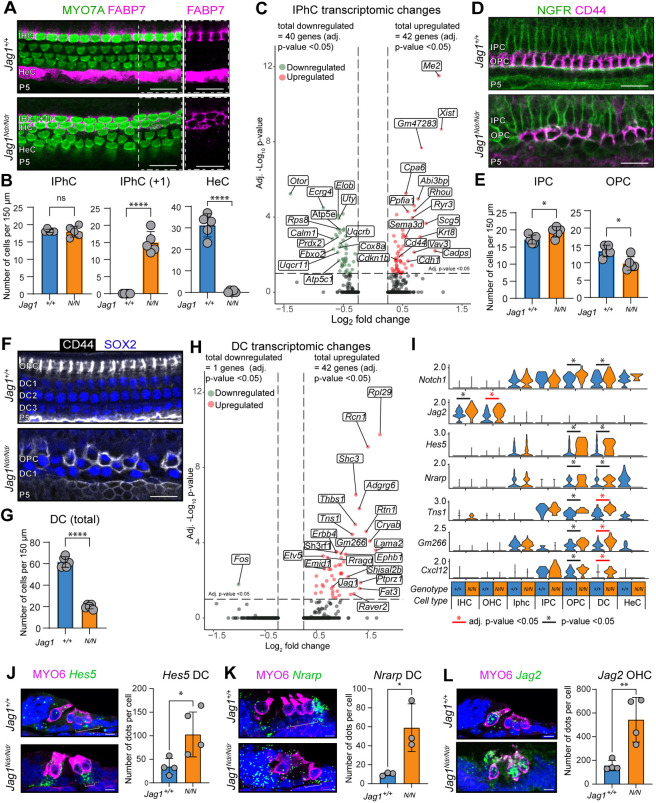
**P5 *Jag1^Ndr/Ndr^* mice exhibit supernumerary IPhCs, fewer lateral SCs, HeC ablation and increased Notch activation in lateral SCs.** (A) HC, IPhC and HeC phenotype at P5, showing a duplication of IHCs (MYO7A, green) and IPhCs (FABP7, magenta) in *Jag1^Ndr/Ndr^* cochleae*.* Right panel shows FABP7 alone, highlighting the lack of FABP7 signal in the lower *Jag1^Ndr/Ndr^* HeC region. (B) Quantification of IPhCs, supernumerary IPhCs (IPhC +1) and HeCs, showing: 18.0±1.6 IPhCs cells per 150 µm in *Jag1^Ndr/Ndr^* compared with 18.2±0.5 in *Jag1^+/+^* (not significant); 15.0±3.2 with supernumerary IPhCs cells (‘IPhC +1’) per 150 µm in *Jag1^Ndr/Ndr^* compared with 0.0±0.0 in *Jag1^+/+^* (****P<0.0001; data are mean±s.d.); and 0.4±0.6 HeC cells per 150 µm in *Jag1^Ndr/Ndr^* compared with 31.2±5.4 in *Jag1^+/+^* (****P<0.0001; data are mean±s.d.). (C) Volcano plot depicting up- and downregulated genes (adj. *P*-value<0.05) in P5 *Jag1^Ndr/Ndr^* IPhCs. (D) PC phenotype, showing IPCs (NGFR, green) and OPCs (CD44, magenta). (E) P5 quantification of IPCs and OPCs in the mid-region, demonstrating an increase in IPCs and a decrease in OPCs, with 19.4±1.5 IPC cells per 150 µm in *Jag1^Ndr/Ndr^* compared with 17.2±1.3 in *Jag1^+/+^* (*P*=0.05), 9.8±1.9 OPC cells per 150 µm in *Jag1^Ndr/Ndr^* compared with 13.4±1.6 in *Jag1^+/+^* (*P<0.05, data are mean±s.d.). (F) DC phenotype staining for DCs (SOX2, blue) and OPCs (CD44, white) demonstrates a reduction of DCs lateral from OPCs. (G) Quantification of DCs demonstrates a decrease in DCs in *Jag1^Ndr/Ndr^* mice, with 20.0±2.1 DC cells per 150 µm in *Jag1^Ndr/Ndr^* compared with 61.6±4.9 in *Jag1^+/+^* (****P<0.0001, data are mean±s.d.). (H) Volcano plot depicting up- and downregulated genes (adj. *P*-value<0.05) in *Jag1^Ndr/Ndr^* DCs. (J) Violin plots depicting the expression of Notch components and of genes identified by Castel et al. (2013) to contain Rbpj-binding sites per genotype and cell type, indicating increased Notch activation in OPCs and DCs. (J) Expression of *Hes5* RNA (green) and myosin 6 (magenta) in *Jag1^+/+^* and *Jag1^Ndr/Ndr^* by RNAscope (left) and quantification (right) showing upregulation of *Hes5* in DCs in *Jag1^Ndr/Ndr^* animals (brackets). (K) Expression of *Nrarp* RNA (green) and myosin 6 (magenta) in *Jag1^+/+^* and *Jag1^Ndr/Ndr^* by RNAscope (left) and quantification (right) showing upregulation of *Nrarp* in DCs in *Jag1^Ndr/Ndr^* mice (brackets), (L) Expression of *Jag2* RNA (green) and myosin 6 (magenta) in *Jag1^+/+^* and *Jag1^Ndr/Ndr^* by RNAscope (left) and quantification (right) showing upregulation of *Jag2* in OHCs in *Jag1^Ndr/Ndr^* mice. Scale bars: 20 µm in A,D,F; 10 µm in J-L. *n*=5 per genotype for phenotype characterization and each dot represents one animal; *n*≥3 or more per quantifications in J-L, dots representing the average of five or more sections per animal. In I, red asterisks indicate significant upregulation (adj. *P*-value<0.05); black asterisks indicates potential upregulation (*P*-value<0.05). *P<0.05; **P<0.01, ****P<0.0001 (unpaired *t*-test).

*Jag1^Ndr/Ndr^* mice exhibited a duplicated row of IHCs, accompanied by a duplication of FABP7^+^ IPhCs (Fig. 3A,B**)**, indicating a medial boundary defect that includes a proportional duplication of both IHCs and IPhCs (Basch et al., 2016). Differential gene expression analysis identified 42 up- and 40 downregulated genes in *Jag1^Ndr/Ndr^* versus *Jag1^+/+^* IPhCs, with pathway dysregulation similar to the pseudo-bulk analyses (Fig. 3C, Tables S5, S6). To study cell identity, we manually curated a list of 200 genes specific for each cell type based on gene expression in the *Jag1^+/+^* dataset, resulting in signature genes for each cell type (Table S7). Downregulated genes in *Jag1^Ndr/Ndr^* IPhCs did not include IPhC signature genes, indicating that *Jag1^Ndr/Ndr^* IPhC identity was not affected by JAG1 insufficiency.

FABP7 (a Notch target gene also known as *Blbp*; Anthony et al., 2005) is a marker of IPhCs, as well as HeCs (Chrysostomou et al., 2020; Kolla et al., 2020), which are present lateral to the OHCs in the *Jag1^+/+^* organ of Corti (Fig. 3A, right panel). However, FABP7 expression was almost completely absent in the lateral compartment along the entire length of the cochlea in *Jag1^Ndr/Ndr^* mice (Fig. 3A,B), and we only sporadically found a FABP7^+^ HeC (Fig. S2A,B). FABP7 absence in the lateral compartment could reflect Notch silencing in HeCs, rather than a de facto loss of HeCs. However, 43 *Jag1^+/+^* and only three *Jag1^Ndr/Ndr^* HeCs were retrieved in the scRNA seq (Fig. 2B, Table S8), corroborating that HeCs are depleted in *Jag1^Ndr/Ndr^* mice.

Next, we assessed PCs, a specialized SC subtype located between IHCs and OHCs whose differentiation and maintenance are Notch-independent (Doetzlhofer et al., 2009). PCs express nerve growth factor receptor (NGFR, also known as p75^ntr^) (Després et al., 1991; Gestwa et al., 1999; Von Bartheld et al., 1991) and outer PCs (OPCs) begin to express CD44 between P0 and P7 (Hertzano et al., 2010). There was an increase in NGFR^+^ PCs and a decrease in CD44^+^ OPCs in the mid region at P5 (Fig. 3D,E), while IPC and OPC numbers were similar in the base region (Fig. S2C,D), indicating that PC development may be delayed in *Jag1^Ndr/Ndr^* mice. We therefore assessed the PC phenotype at birth. As expected, *Jag1^+/+^* mice exhibited one row of NGFR^+^ IPCs accompanied by one row of CD44^+^ OPCs. However, *Jag1^Ndr/Ndr^* mice showed a delay in PC development, displaying a more widespread NGFR^+^ signal reaching two to three cell layers, and almost completely lack CD44^+^ cells, mimicking a less mature phenotype that is typically observed around E17.5 in *Jag1^+/^*^+^ mice (Fig. S2C). Despite the developmental delay observed in IPCs and OPCs, only minor transcriptomic differences were detected in IPCs and OPCs between *Jag1^Ndr/Ndr^* and *Jag1^+/+^* animals, with only one gene, *Rpl29*, being upregulated in OPCs (adj. *P*-value<0.05, Table S5).

Deiter's cells (DCs) were severely reduced in *Jag1^Ndr/Ndr^* mice (Fig. 3F,G), with a total of 30 cells compared with 126 cells in the *Jag1^+/+^* dataset (Table S8). Differential gene expression analysis identified 42 up- and one downregulated genes in *Jag1^Ndr/Ndr^* DCs versus *Jag1^+/+^* DCs (Fig. 3H, Table S5, S7), but there was no difference in DC signature genes (Table S7), suggesting remnant *Jag1^Ndr/Ndr^* DCs are correctly specified.

The reduction in lateral SCs, and lack of HeCs, could be a consequence of disrupted prosensory domain establishment in the absence of *Jag1*. At E14.5, *Jag1^Ndr/Ndr^* mice exhibited similar SOX2^+^ and JAG1^+^ domains as *Jag1^+/+^* mice (Fig. S3A,B) but smaller lateral SOX2^+^JAG1^−^ lateral prosensory domains, and reduced expression of the Jag1-dependent prosensory domain marker CDKN1B (Fig. S3A,B). Corroborating an overall reduction in Notch target gene expression during prosensory domain establishment, RNAscope demonstrated reduced expression of the Notch target genes *Heyl*, *Hey1*, *Hey2* and *Hes1* in this domain (Fig. S3). In summary, a smaller initial *Jag1^Ndr/Ndr^* lateral prosensory domain may limit development of lateral DCs and HeCs.

### scRNAseq analysis demonstrates increased Notch activation in *Jag1^Ndr/Ndr^* lateral SCs

To study Notch activation status, we next investigated Notch component and target gene expression in *Jag1^Ndr/Ndr^* and *Jag1^+/+^* SCs and HCs. Supporting cells, including DCs, expressed both Notch receptors and ligands, including *Notch1* and *Jag1*, with a tendency towards upregulation in *Jag1^Ndr/Ndr^* DCs (Fig. 3I, Fig. S4A). *Jag1^Ndr/Ndr^* DCs also exhibited robust expression of Notch target genes *Hes5* and *Nrarp*, and their upregulation in *Jag1^Ndr/Ndr^* DCs was corroborated by RNAscope (Fig. 3J,K).

To further study the Notch activation status of SCs, we cross-referenced differentially expressed genes in lateral SCs (IPCs, OPCs and DCs, avg. log_2_ FC>0.25 or <−0.25 and *P*-value<0.05) with genes previously reported to contain inducible binding sites for the Notch transcription factor Rbpj (Castel et al., 2013). Of 258 reported target genes (Castel et al., 2013), 27 were, in addition to *Jag1*, *Notch1* and *Nrarp*, differentially expressed in *Jag1^Ndr/Ndr^* lateral SCs, of which most (20) were upregulated, corroborating increased Notch activation in lateral *Jag1^Ndr/Ndr^* SCs (Fig. 3I, Fig. S4B). In contrast to lateral SCs, HeCs do not express *Jag1*, but conditional deletion of *Jag1* results in the loss of HeCs (Chrysostomou et al., 2020). Although the statistical power is limited due to the low HeC number in *Jag1^Ndr/Ndr^* mice, the few remaining *Jag1^Ndr/Ndr^* HeCs downregulated the Notch target gene *Nrarp* (Fig. 3I), suggesting lateral SCs (OPC and DCs) and HeCs do not respond similarly to *Jag1* insufficiency. In line with this, most of the Notch-inducible genes were upregulated in lateral SCs, but downregulated in HeCs (Fig. 3I). Finally, *Jag2* was significantly upregulated in *Jag1^Ndr/Ndr^* OHCs (Fig. 3I,L, Table S5), suggesting that *Jag1* represses *Jag2* expression in OHCs. In summary, Notch signaling was activated in *Jag1^Ndr/Ndr^* lateral SCs, indicating a role for Jag1 in limiting Notch activity in lateral SCs, either directly via cis-inhibition within supporting cells, or indirectly by limiting Jag2 expression and trans-activation from HCs.

### scRNAseq analysis of *Jag1^Ndr/Ndr^* HCs reveals a role for Jag1 in OHC development and function

**Fig. 4.**
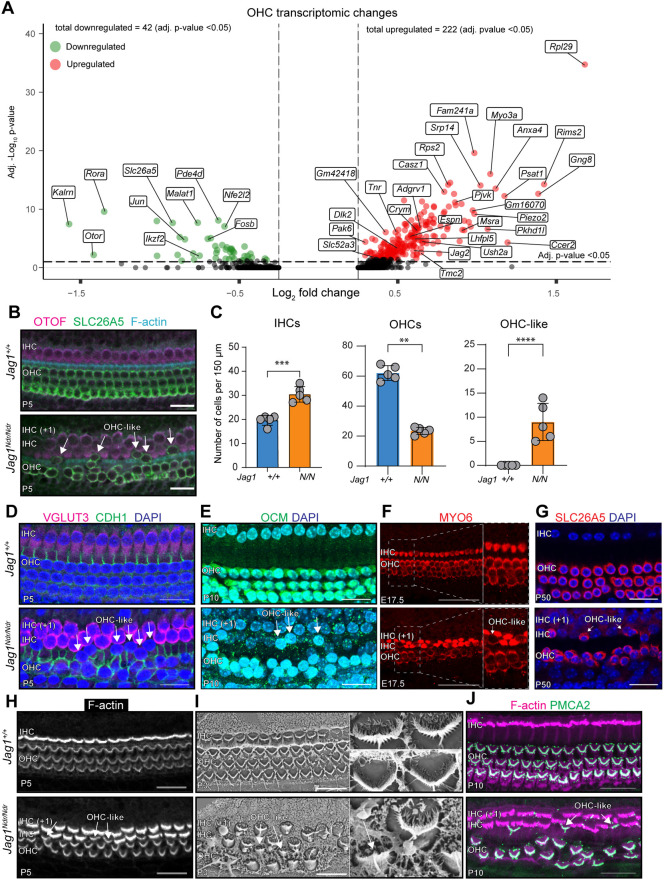
***Jag1^Ndr/Ndr^* OHCs exhibit major transcriptomic dysregulation and HC patterning defects.** (A) Volcano plot depicting downregulated (left, green) and upregulated genes (right, red) for *Jag1^Ndr/Ndr^* OHCs (adj. *P*-value<0.05). (B) HC phenotype, showing IHCs (OTOF, magenta) and OHCs (SLC26A5, green), and PC protrusions (F-actin, blue), showing a duplication of IHCs, fewer lateral OHCs and the presence of SLC26A5-positive cells outside the lateral OHC domain, referred to as OHC-like cells (white arrows). (C) Quantification of IHCs, OHCs and OHC-like cells: 30.4±3.2 IHCs per 150 µm in *Jag1^Ndr/Ndr^* compared with 19.6±2.1 in *Jag1^+/+^* (***P<0.001), 23.4±2.4 total OHCs per 150 µm in *Jag1^Ndr/Ndr^* compared with 62.0±5.0 in *Jag1^+/+^* (**P<0.0001), 9.0±1.7 OHC-like cells per 150 µm in *Jag1^Ndr/Ndr^* compared with 0.0±0.0 in *Jag1^+/+^* (****P<0.001). Data are mean±s.d. (D) Phenotypic characterization of HCs in *Jag1^Ndr/Ndr^* mice (lower panels) at various stages of development showing IHCs (VGLUT3, magenta) and E-cadherin (CDH1, green), indicating absent IHC marker expression in OHC-like cells (white arrows), and the presence of OHC-like cells outside the E-cadherin positive domain. (E) Oncomodulin (OCM, green) expression in OHC-like cells at P10. (F) E17.5 cochleae stained for HCs (MYO6, red), demonstrating the presence of OHC-like cells in the inner/medial compartment, based on morphological characteristics. (G) OHCs (SLC26A5, red) at P50 (adult mice), with OHC-like cells in *Jag1^Ndr/Ndr^* mice. (H) Assessment of stereocilia (F-actin, white), using a different focal plane from the same cochleae as in B, demonstrating that *Jag1^Ndr/Ndr^* OHC-like cells display V-shaped stereocilia and similar F-actin intensity similar to OHCs. (I) Scanning electron microcopy (SEM) images of stereocilia, confirming OHC-like cell stereocilia resemble OHC stereocilia. (J) OHC stereocilia-specific PMCA2 staining (green), demonstrating PMCA2-positive stereocilia outside the lateral compartment in *Jag1^Ndr/Ndr^* mice. *n*=5 per genotype; data are mean ±s.d. Scale bars: 20 µm in B,D,E,G-J; 50 µm in F. **P<0.01; ***P<0.001, ****P<0.0001 (unpaired *t*-test).

Finally, to determine whether Jag1 controls IHC or OHC specification, we analyzed *Jag1^Ndr/Ndr^* and *Jag1^+/+^* IHC and OHC transcriptomes. IHC transcriptomes were similar between *Jag1^Ndr/Ndr^* and *Jag1^+/+^* animals, with only five significantly dysregulated genes (Table S5). Cross-referencing an extended list of *Jag1^Ndr/Ndr^* IHC putatively dysregulated genes (322 genes, *P*-value<0.05) with the curated IHC signature (Table S7), revealed that only four out of 322 genes (1.2%) were IHC-enriched. Additional subsetting, renormalization and subclustering of hair cells did not reveal any additional IHC populations in the *Jag1^Ndr/Ndr^* dataset, indicating that extra IHCs (+1 IHCs) are transcriptionally similar to IHCs, and do not separate out as an additional population (Fig. S5A,B). Therefore, even in presence of a medial border defect resulting in supernumerary IHCs and IPhCs (Figs 3A,B, 4B,C), the reduced Jag1 signaling does not significantly perturb IHC identity.

In contrast to IHCs, *Jag1^Ndr/Ndr^* OHCs exhibited highly dysregulated gene expression, with 222 up- and 42 downregulated genes (Fig. 4A). Cross-referencing *Jag1^Ndr/Ndr^* OHC dysregulated genes (264 genes, adj. *P*-value<0.05) with the curated OHC signature (Table S7), revealed that 40 of the dysregulated genes (15.2%) were OHC-enriched. In addition to *Jag2*, Notch pathway components *Dlk2* and *Hes6* were upregulated, as well as genes associated with inner ear and stereocilia development (Fig. 4A). Downregulated genes included mature OHC markers *Ikzf2* and *Slc26a5* (Fig. 4A). Subsetting and renormalizing the HC subpopulation corroborated mature OHC marker downregulation, including *Ocm*, and upregulation of immature OHC makers such as *Bcl11b* and *Insm1* (Fig. S5C, Table S9)*.* In summary, *Jag1^Ndr/Ndr^* showed major transcriptomic dysregulation in OHCs, indicating a role for Jag1 in OHC gene regulation and development.

### *Jag1^Ndr/Ndr^* mice exhibit OHCs in the IHC and PC compartments

Given the major transcriptomic changes in *Jag1^Ndr/Ndr^* OHCs, we examined HC patterning in greater detail. Whereas *Jag1^+/+^* cochleae displayed the conserved one row of IHCs and three rows of OHCs, *Jag1^Ndr/Ndr^* mice exhibited a complete second row of supernumerary IHCs (+1 IHC) (Fig. 4B-D, Fig. S6A), and the number of OHCs was greatly reduced, with an absent third OHC row (Fig. 4B,C). *Jag1^+/Ndr^* mice exhibited an intermediate phenotype with the occasional supernumerary IHC, and occasional missing OHCs in the third OHC row (Fig. S6B), suggesting a dose-dependent IHC and OHC phenotype. Interestingly, *Jag1^Ndr/Ndr^* mice exhibited sporadic ectopic expression of the OHC marker SLC26A5^+^ (Prestin) in HCs in the IHC and PC compartments (Fig. 4B, white arrows). As these cells exhibited OHC marker expression, but were found outside the OHC region, we refer to these ectopic cells as ‘OHC-like’ cells. OHC-like cells were exclusively observed in *Jag1^Ndr/Ndr^* mice but never in *Jag1^+/+^* or *Jag1^+/Ndr^* animals (Fig. 4C, Fig. S6B-D). The *Jag1^Ndr/Ndr^* phenotype was consistent along the entire length of the cochlea (Fig. S6E).

To further characterize OHC-like cells, we assessed their expression of an array of IHC- and OHC-specific markers. OHC-like cells were negative for the IHC markers otoferlin (OTOF) and vesicular glutamate transporter 3 (VGLUT3), as well as the outer compartment marker E-cadherin (CDH1) (Fig. 4D) but expressed the OHC marker oncomodulin (OCM) (Fig. 4E). OHC-like cells were identifiable, based on morphological characteristics, as early as embryonic day (E) 17.5 (Fig. 4F, Fig. S6B) and were still present after complete differentiation and maturation of the cochlea at P50 (Fig. 4G). We next asked whether OHC-like cells would separate out as an additional cell population in the scRNAseq dataset. Renormalizing and unsupervised reclustering of the HC dataset did reveal an OHC cell population exclusive to *Jag1^Ndr/Ndr^* mice, with high expression of *Sez6l* and *Bmp2* (Fig. S7). However, RNAscope validation of *Sez6l* and *Bmp2* RNA expression demonstrated expression of these genes in PCs, rather than OHCs for both *Jag1^Ndr/Ndr^* and *Jag1^+/+^* animals (Fig. S7). Given the location of some of the OHC-like cells in the PC compartment, they may be subject to a greater risk of contamination with PC detritus during dissection and dissociation. Removing a PC gene signature (80 genes found in *Jag1^Ndr/Ndr^* OHCs and in PCs of any genotype, Table S9) eliminated the separation of the *Jag1^Ndr/Ndr^* OHC subpopulation, suggesting that the separation of this population could be driven by PC contamination, but also indicating that the OHC population of the *Jag1^Ndr/Ndr^* dataset likely contains both OHC-like cells located in the PC region and de facto OHCs, which are indistinguishable in this dataset in the absence of the PC signature (Fig. S7E, Table S9). The upregulation of immature OHC gene expression remained after PC signature removal, but pseudotime analysis indicated no statistically significant difference in pseudotime between *Jag1^Ndr/Ndr^* and *Jag1^+/+^* OHCs (Fig. S8), indicating that the developmental delay of *Jag1^Ndr/Ndr^* OHCs, if any, is modest*.*

To further ascertain whether the ectopic OHC-like cells recapitulate the morphological and functional characteristics of OHCs, we next investigated morphology and molecular characteristics of stereocilia. All *Jag1^Ndr/Ndr^* HCs were able to take up the FM1-43 dye, indicating that transduction channels in *Jag1^Ndr/Ndr^* stereocilia are functional (Fig. S9). Moreover, the stereocilia of OHC-like cells had a V-shaped arrangement as well as low intensity F-actin staining, resembling *Jag1^+/+^* OHCs (Fig. 4H). Scanning electron microscopy (SEM) demonstrated a sparse arrangement of HCs in *Jag1^Ndr/Ndr^* mice, with OHC-like cells exhibiting stereocilia bundle arrangement and characteristics of OHCs (Fig. 4I). Finally, stereocilia of OHC-like cells were positive for the OHC stereocilia marker plasma membrane calcium pump Ca2^+^-ATPase 2 (PMCA2) (Chen et al., 2012) (Fig. 4J). Altogether, OHC-like cells express markers and morphology of bona fide OHCs, including hallmarks of wild-type OHC stereocilia. Furthermore, a *Jag1^Ndr/Ndr^* OHC sub-population, identified by a PC signature, clusters out upon sub-setting and re-clustering of the HC population.

### OHC-like cells exhibit a location-dependent expression of *Tbx2*

**Fig. 5.**
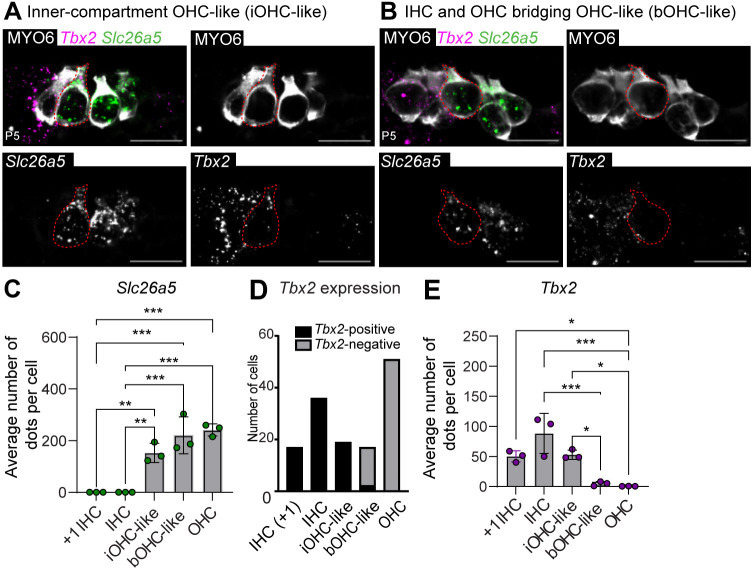
***Jag1^Ndr/Ndr^* OHC-like cells exhibit location-dependent expression of the IHC fate determinant *Tbx2.*** (A,B) RNAscope for mRNA expression of the IHC regulator *Tbx2* (magenta) and OHC marker *Slc26a5* (green) in inner (A) and bridging (B) OHC-like cells, with HC counterstain (MYO6, white), demonstrating *Tbx2* and *Slc26a5* positivity in inner OHC-like cells and no *Tbx2* expression in bridging OHC-like cells (red encircled cells). (C) Quantification of *Slc26a5* puncta in *Jag1^Ndr/Ndr^* HC subtypes, where each dot represents the average of five or more sections quantified per animal. (D) Positivity of HC subtypes for *Tbx2* (binary yes/no). (E) Quantification of *Tbx2* mRNA puncta in *Jag1^Ndr/Ndr^* HC subtypes, where each dot represents the average of five or more sections quantified per animal. *n*=3 per genotype; data are mean±s.d. Scale bars: 20 µm. *P<0.05; **P<0.01; ***P<0.001 (one-way ANOVA with Bonferroni correction).

Factors determining an IHC versus OHC fate have recently been identified (Bi et al., 2022; García-Añoveros et al., 2022; Kaiser et al., 2022; Li et al., 2023; Wiwatpanit et al., 2018), but it is not known whether Notch signaling controls expression of these genes in HC subtypes. We therefore investigated recently identified factors specifying these cell fates, focusing on the IHC determinant *Tbx2* (García-Añoveros et al., 2022). We predicted that *Jag1^Ndr/Ndr^* OHC-like cells should not express *Tbx2*, as its overexpression is sufficient to induce an IHC fate in OHCs, and ablation of *Tbx2* induces trans differentiation of IHCs to OHCs (García-Añoveros et al., 2022). However, *Tbx2* was specifically expressed by the OHC-like cells found between IHCs (iOHC-like), but not by OHC-like cells bridging the IHC and OHC compartment, between PCs (bOHC-like) (Fig. 5, Fig. S10). In the presence of both *Tbx2* and *Slc26a5*, *Jag1^Ndr/Ndr^* HCs are OHC like, demonstrating that the expression of *Tbx2*, at least at the levels expressed in OHC-like cells, is not sufficient to consolidate an IHC fate in the *Jag1^Ndr/Ndr^* cochlea.

In summary, *Jag1^Ndr/Ndr^* mice exhibit atypical OHC-like cells in the IHC and PC compartments, in addition to supernumerary IHCs and a reduction in OHCs. OHC-like cells demonstrated a position-dependent expression of the IHC fate-determinant *Tbx2*, indicating that defective Jag1 signaling renders some HCs insensitive to the IHC fate determinant *Tbx2*.

## DISCUSSION

In this study, we used the Nodder mouse model of Alagille syndrome, to address how *Jag1* insufficiency affects individual SC and HC subtypes at single cell resolution. *Jag1^Ndr/Ndr^* mice exhibited defects in prosensory domain size and Notch activation, medial boundary formation and lateral compartment defects. Importantly, OHC-like cells were present in the IHC or PC compartments and were insensitive to the IHC-determinant *Tbx2*, shedding new light on the potency of *Tbx2* in IHC fate determination in the context of defective Notch signaling. Together, our data demonstrate that *Jag1^Ndr/Ndr^* mice provide a model for hearing loss in ALGS and provide high-resolution insights into medial and lateral compartment development in the *Jag1*-compromised disease state.

### Boundary formation and lateral inhibition

Notch signaling establishes a boundary between the organ of Corti and Kolliker's organ (Basch et al., 2016), and patterns HCs and SCs via lateral inhibition. Partial reduction in Notch signaling results in a medial boundary defect with a proportional duplication of both a SC and a HC, while complete abrogation of Notch signaling results in both a boundary cell defect and profound lateral inhibition defects with a large excess of IHCs and no IPhCs (Basch et al., 2016). Nodder mice exhibited a dose-dependent medial boundary defect that included a slight increase in IHCs in *Jag1^+/Ndr^* mice (Fig. S5), and a full duplication of both IHCs and IPhCs in *Jag1^Ndr/Ndr^* mice (Figs 3, 4). The duplication and persistence of IPhCs show that lateral inhibition (by Dll1 or Jag2) is not lost in the *Jag1^Ndr/Ndr^* medial domain. Similarly, in the lateral domain, the size of which is likely restricted due to hampered prosensory domain establishment (Fig. S2), the reduction in OHCs was accompanied by a proportional reduction in the associated DC population, again suggesting preserved lateral inhibition. Interestingly, Notch signaling is specifically required for DC development and survival (Campbell et al., 2016), in which case DCs could be expected to be more severely reduced than OHCs in *Jag1^Ndr/Ndr^* mice. Instead, *Jag1^Ndr/Ndr^* DCs were reduced in proportion to the OHC reduction and exhibited a NOTCH-ON signature, with upregulation of multiple Notch target genes (Fig. 3I-K). The increased Notch activation in *Jag1^Ndr/Ndr^* SCs can be explained by two modes of Notch activation: (1) trans-activation from increased expression of Jag2 in *Jag1^Ndr/Ndr^* OHCs (Fig. 4A); or (2) failure of JAG1^NDR^ to mediate Notch *cis*-inhibition within SCs. Previous work has shown that medial SCs can experience *cis*-inhibition (Basch et al., 2016). However, data presented here suggest JAG1 might *cis*-inhibit Notch signaling in DCs but does not provide evidence for Jag1 *cis*-inhibited Notch signaling in IPhCs. The JAG1^NDR^ missense mutant is expressed *in vivo*, and traffics normally (Hansson et al., 2010), but does not bind or activate NOTCH1 (Andersson et al., 2018). Our data do not allow us to conclusively determine whether Jag1 *cis*-inhibition or Jag2 *trans*-activation mediates the observed Notch activation in lateral *Jag1^Ndr/Ndr^* SCs but indicates that great care must be taken to consider the levels of all Notch receptors and ligands in all cochlear cell types in these types of analyses. Experiments reducing *Jag2* levels in *Jag1^Ndr/Ndr^* OHCs, by reducing *Jag2* gene dosage in *Jag1^Ndr/Ndr^* mice or by using JAG2 blocking antibodies, could theoretically address whether *Jag1^Ndr/Ndr^* SC Notch activation is dependent on *Jag2* upregulation in OHCs, but would be technically challenging due to the required specific mixed genetic background of *Jag1^+/Ndr^* mice (Andersson et al., 2018), their vascular frailty (Hankeova et al., 2022) and their high mortality (Andersson et al., 2018; Hankeova et al., 2022). Additionally, conditional deletion of Jag1 at various timepoints could provide valuable insights into the distinct roles for Jag1, which our model does not allow us to study, as the JAG1^NDR^ missense mutation is constitutive.

### Origin of OHC-like cells

The *Jag1^Ndr/Ndr^* model is a *Jag1* insufficiency model (Andersson et al., 2018; Hansson et al., 2010). The JAG1^NDR^ protein cannot bind or activate NOTCH1, but displays some residual binding and activation of NOTCH2, and can bind NOTCH3. However, an unknown neomorphic function for JAG1^NDR^ cannot be excluded. While OHC-like cells have not previously explicitly been described in the inner hair cell compartment of *Jag1*-compromised mice, two other *Jag1* missense mutant mice – Slalom (Slm, *Jag1^+/Slm^*) and Headturner (Htu, *Jag1^+/Htu^*) mice – display atypical hair cells in the IHC compartment with OHC stereocilia features (Kiernan et al., 2001; Tsai et al., 2001). The presence of atypical OHC-like cells in the IHC compartment are thus likely a product of dysregulated Jag1 signaling, rather than a consequence of neomorphic Jag1 function in *Jag1^Ndr/Ndr^* mice. However, rather than being placed lateral to the bona fide IHCs, as the *Jag1^Ndr/Ndr^* OHC-like cells are, the Slm and Htu atypical hair cells are placed medial to the IHC row, demonstrating that the placement of the extra OHCs is likely sensitive to the nature of the *Jag1* perturbation.

OHC and IHC specification in the lateral and medial compartments, respectively, is a tightly regulated process, for which only a few perturbations have recently been described (Bi et al., 2022; Chessum et al., 2018; García-Añoveros et al., 2022; Kaiser et al., 2022; Li et al., 2023; Wiwatpanit et al., 2018). The presence of OHC-like stereocilia on both iOHC-like cells and bOHC-like cells, including the V-shape bundle arrangement, F-actin intensity levels and PMCA2 positivity argue against a direct IHC-to-OHC-like cell conversion (Fig. 4H-J). Conditional deletion of *Tbx2* induces neonatal transdifferentiation of IHCs into OHCs, expressing several OHC molecular markers but lacking OHC stereocilia features. Instead, the transdifferentiated cells maintained stereocilia that resemble the overall appearance of IHC stereocilia (García-Añoveros et al., 2022). HC stereocilia develop during a limited time window during a late embryonic and early postnatal period and do not alter stereocilia size and bundle arrangement once stereocilia are formed and are not replaced once lost (Jia et al., 2009; Sekerková et al., 2011). This indicates that *Jag1^Ndr/Ndr^* OHC-like cells most likely were born as OHCs rather than converted from IHCs into OHC-like cells. Although our experiments do not directly address the developmental origin of the *Jag1^Ndr/Ndr^* OHC-like cells, published data, their anatomical location and transcriptomic data suggest that bOHC-like cells might originate from PCs or their precursors. Ectopic HCs in the PC region are present at the position of missing PCs in *Hey1, Hey2* double mutant mice, suggesting a late PC-to-HC conversion (Benito-Gonzalez and Doetzlhofer, 2014). *Jag1^Ndr/Ndr^* OHC-like cells appear before IPC and OPC differentiation, and PC differentiation is delayed in *Jag1^Ndr/Ndr^* mice. Based on the position of the bOHC-like cells, the PC precursor population could be a source of cells that differentiate into OHC-like cells. However, SCs that convert into HCs typically retain at least a partial SC gene expression signature, including low levels of SOX2 (Iyer et al., 2022), which we did not observe at various stages using SOX2 immunostaining, nor in any of the scRNAseq OHC populations. Nonetheless, OHC subsetting revealed a *Jag1^Ndr/Ndr^*-specific OHC population expressing a PC signature (Fig. S7), which could reflect either contamination of *Jag1^Ndr/Ndr^* cells specifically due to their anatomical location or a divergent developmental origin. Therefore, OHC-like *Jag1^Ndr/Ndr^* cells are more likely to have originated from PCs, or from a PC precursor pool, than from IHCs, and future studies using, for example, time-lapse imaging of developing *Jag1^Ndr/Ndr^* cochlear explants or lineage tracing could help resolve whether iOHCs and bOHCs share a developmental origin, and whether these arise from IHCs, PCs or other cells.

### Context of cell fate determinants

Recently, the identification of IHC- versus OHC-specific factors, including *Tbx2* (Bi et al., 2022; García-Añoveros et al., 2022; Kaiser et al., 2022), *Insm1* (Li et al., 2023; Wiwatpanit et al., 2018) and *Ikzf2* (Chessum et al., 2018), have significantly contributed to understanding HC divergence into IHC and OHCs, and highlighted neonatal plasticity between the two cell types. Although an interplay between Notch and these factors have been suggested in other tissues (Jia et al., 2015; Rutenberg et al., 2006), it has not been reported whether Notch signaling regulates these factors in the cochlea. *Tbx2* is epistatic to *Insm1* (Li et al., 2023): in the absence of both *Tbx2* and *Insm1*, only OHCs develop, demonstrating that *Tbx2* is required for normal IHC differentiation and trans-differentiation of Insm1-deficient OHCs into IHCs. Ectopic *Tbx2* expression in P0 cochlear explant OHCs represses OHC features, including Prestin expression, and induces IHC features, including VGLUT3 expression (García-Añoveros et al., 2022). However, *Jag1^Ndr/Ndr^* mice exhibited *Tbx2*^+^ iOHC-like cells, indicating that *Tbx2* is not sufficient to induce an IHC fate in iOHC-like cells in the absence of functional Jag1. Importantly, *Tbx2* is an organ of Corti inner compartment patterning factor (Kaiser et al., 2022), which may explain its expression in *Jag1^Ndr/Ndr^* OHC-like cells in the IHC compartment. It is possible that Tbx2 dosage is important for consolidating the IHC fate: *Tbx2* levels were lower in iOHC-like cells compared with IHCs, which might permit the OHC-like phenotype (Fig. 5E). However, the supernumerary IHCs (IHC+1) expressed similar levels of *Tbx2* as iOHC-like cells but developed as IHCs rather than OHCs (Fig. 5E), arguing against Tbx2 gene dosage as the sole determining factor in *Jag1^Ndr/Ndr^* OHC-like phenotype. More recently, it has been shown that *Tbx2* overexpression alone is insufficient to induce an IHC fate in medial SCs (Bi et al., 2022), indicating that further studies are required to understand the function of Tbx2 in driving an IHC fate in different contexts.

### Hearing and balance in Alagille syndrome

Both conductive and sensorineural hearing loss are reported in individuals with Alagille syndrome (Teng et al., 2017), as well as vestibular defects (Okuno et al., 1990). Similarly, *Jag1*-deficiency results in hearing loss in mice (Chrysostomou et al., 2020; Gilels et al., 2022; Kiernan et al., 2006; Vrijens et al., 2006) (Table S2), and *Jag1* missense mutant mice are nicknamed for overt balance defects, including *Headturner* (Kiernan et al., 2001), *Ozzy* (Vrijens et al., 2006) and *Slalom* (Tsai et al., 2001). ABR measurements indicated severe hearing loss in *Jag1^Ndr/Ndr^* mice, as well as reduced cochlear amplification assessed by DPOAE. Additionally, there were major transcriptomic changes in OHCs in *Jag1^Ndr/Ndr^* mice, indicating that the OHC function of remaining OHCs might be compromised.

As a constitutive model, *Jag1^Ndr/Ndr^* mice present pathologies in multiple hearing organs that can contribute to hearing loss. Previous work (Chrysostomou et al., 2020; Gilels et al., 2022; Teng et al., 2017; Vrijens et al., 2006) explored how specific *Jag1*-related inner ear pathologies can contribute to hearing deficits. Heterozygous *Jag1* mutant models display only mild hearing loss (Kiernan et al., 2006; Tsai et al., 2001; Vrijens et al., 2006), while malformation of the stapes, induced by neural crest-specific *Jag1* loss of function, results in moderate-to-severe elevated ABR thresholds (+30 dB) along all frequencies (Teng et al., 2017). Deletion of *Jag1* in SCs at birth, using *Fgfr3-iCreER^T2^*, abrogates Hensen's cell formation and maintenance, and results in hearing deficits in the low-frequency range (Chrysostomou et al., 2020). SC-specific deletion of *Jag1* at birth, with *Sox2-Cre^ERT2^*, causes IHC stereocilia defects, but no OHC/DPOAE defects, with a circa 40 dB ABR threshold shift along all frequencies (Gilels et al., 2022). The severe hearing loss in *Jag1^Ndr/Ndr^* mice might therefore result from the combination of middle ear bone malformations (stapes defect), patterning defects (loss of OHCs and DCs) and functional compromise of remaining cells (major transcriptomic changes in OHCs), reflecting both conductive and sensorineural hearing loss. The work reported here, and previous work, therefore suggests that each defect likely contributes to worsening hearing loss in *Jag1^Ndr/Ndr^* mice, which could therefore serve as a model to study mixed hearing loss and test possible therapeutic interventions in Alagille syndrome.

Phenotype presentation in ALGS is generally diverse, and there is no reported genotype-phenotype correlation. Although features of ALGS such as intrahepatic bile duct paucity and vascular defects similarly exhibit variability in *Jag1^Ndr/Ndr^* mice (Andersson et al., 2018; Hankeova et al., 2021, 2022), the inner ear defects were remarkably consistent within *Jag1^Ndr/Ndr^* and *Jag1^Ndr/+^* mice. The inner ear may thus provide a model that is less sensitive to environmental modifiers, or genetic modifiers in the mixed C3H/C57bl6 background in which the Nodder colony must be maintained. *Jag1^Ndr/Ndr^* mice thus present a relevant disease model in which to test potential therapeutics, and demonstrate that a genotype-phenotype correlation (or dose-dependency) might be present in some organs and absent in others.

### Concluding remarks

In conclusion, this study reveals new functions for *Jag1* in cochlear cell patterning and cell identity, with single cell resolution. In addition to previously reported phenotypes, we identified and characterized a previously unreported *Tbx2* and *Slc26a5* double positive OHC-like cell in the medial compartment, shedding new light on the potency of *Tbx2* in IHC fate determination in the context of defective Notch signaling. The scRNA seq dataset provides an atlas of the Notch-dysregulated cochlea, and reveals major gene dysregulation in OHCs, as well as a previously unreported role for Jag1 regulating Notch activation in lateral SCs. By characterizing patterning defects, as well as transcriptomic changes in *Jag1^Ndr/Ndr^* mice, these data lay a foundation for future investigation of the interaction between Notch signaling and sensory cell patterning and specification, as well as HC divergence and regeneration, and demonstrate that *Jag1^Ndr/Ndr^* mice provide a model for ALGS with hearing and balance defects.

## MATERIALS AND METHODS

### Animals

*Jag1^+/Ndr^* mice originate from the ENU-induced mutation program, as described previously (Hansson et al., 2010). Mice were maintained on a C3H/C57BL6 mixed background as described previously (Andersson et al., 2018). F2 generation mice were used for this study. All experiments were performed in accordance with EU rules and regulations, as approved by the Swedish Board of Agriculture (Jordbruksverket) under ethical permit numbers N5253/2019 and N2987/2020. To obtain timed embryonic and postnatal animals, animals were mated overnight and noon of the day of vaginal plug was considered embryonic day (E) 0.5. Animals were maintained under standard day/night cycles and provided with food and water *ad libitum*, and housed in enriched individually ventilated cages (wood, paper embedding and cardboard tunnels), with a maximal number of six animals per cage. Each mouse used was genotyped by Transnetyx (Cordova, TN, USA). Both male and female mice were used for experiments, with efforts made to maintain equal ratios of sexes when possible. Age of animals varied according to experiment, with specific ages listed in figure panels and in different Materials and Methods subsections. The research equation method was used to determine the appropriate samples size per experiment.

### Open field test – circling analysis

Male and female *Jag1^+/+^* and *Jag1^Ndr/Ndr^* mice (six to eight mice per sex and genotype) at 3-12 months were used for the Open Field Test. Mice were gently placed in a plexiglass open-field box and filmed with a TSE VideoMot2 system. Distance travelled in pixels was converted to actual distance using a scale measure. Each mouse was filmed for 30 min, and the box was cleaned with water and dried in between each mouse. The experimenters were two different female scientists, who sat still during the experiments.

### Auditory measurements

All auditory measurements were performed using Tucker-Davis Technologies System III hardware and software, and stimuli were generated using BioSigRZ software connected to a signal processor (RZ6). For DPOAEs, sound from two independently driven MF1 speakers was merged into a custom acoustic coupler for closed field stimulation. For ABR, the stimulus output was through an open field speaker (MF1). A Brüel and Kjær ¼-inch microphone and a conditioning pre-amplifier (4939 A 011 and 2690 A 0S1) were used to calibrate the stimulus. Speakers were calibrated one at a time using a frequency sweep (4 to 32 kHz). The output was corrected to produce a flat spectrum at 90 dB SPL (open field speaker, ABR) and 80 dB SPL (closed field speakers, DPOAEs). Calibration of speakers was performed daily before auditory measurements. Sex-matched animals from different genotypes in age range P45-P60 were anesthetized using a mixture of ketamine [ketaminol (50 mg/ml), Intervet, 511485] and xylazine [Rompunvet (20 mg/ml), Bayer, KPOCRD5] (100 and 10 mg/kg body weight, respectively) by intraperitoneal injection and placed in a custom-made acoustic chamber. Body temperature was maintained at 36.5°C using a heating pad (Homeothermic Monitoring System 55-7020, Harvard Apparatus) and eye gel was applied to prevent ocular dehydration during anesthesia. DPOAEs were recorded before ABR measurement, by placing an acoustic coupler into the ear canal. A microphone (EK 23103, Knowles) was inserted into the acoustic coupler and connected to a pre-amplifier (ER-10B+, Etymotic Research) and a processor (200 kHz sample rate) to measure sound intensity in the ear canal. Each speaker played one of two primary tones (f1 and f2) and swept in 5 dB steps from 80 to 10 dB SPL (for f2). The 2f1-f2 distortion product was measured with f2=8, 12, 16, 24 and 32 kHz, f2/f1=1.25 and stimulus intensity L1=L2+10 dB SPL. After DPOAE, ABRs were measured by subdermal placement of stainless-steel needle electrodes at the head vertex (positive), under the right ear pinna (negative) and above the right leg (ground). ABRs were evoked by tone bursts (0.5 ms rise per fall time, 5 ms duration) of 8, 12, 16, 24 and 32 kHz presented 21 times per second. Signals were collected via a low-impedance head stage (RA4LI) connected to a pre-amplifier (RA4PA) and digitally sampled (200 kHz sample rate). Responses to 1000 bursts were bandpass filtered at 0.3 to 3 kHz using BioSigRZ and averaged at each intensity. For each frequency, sound intensity decreased from 90-dB SPL in 5-dB steps. Repeated injection of anesthesia, at one quarter of the initial dose, was occasionally given during the ABR measurement to ensure stable anesthesia throughout the measurement. ABR thresholds were determined by the lowest sound stimulus for which a reproducible ABR waveform was identified. DPOAE thresholds were determined as the lowest sound stimulus by which a DPOAE amplitude reached 5 dB above noise.

### Tissue dissociation and library preparation for single cell RNA sequencing

Pups were decapitated at postnatal day 5 and cochleae were collected. The sensory epithelium and surrounding mesenchyme were dissected out. Two cochlea from each pup were incubated for 15 min in 3 ml DMEM:F12 with 100 μl Thermolysin (Sigma, 5 mg/ml) and 5 μl DNase (1 mg/ml, Stemcell Technologies) at 37°C. Cells were dissociated in pre-activated papain (20 U/ml, Worthington Papain Dissociation Kit, LK003150, activated by incubating at 37°C for 30 min with open lid) for a total of 15-20 min with trituration every 5 min using 200 μl low bind tips (Sartorius, 790101F). Papain was inactivated using an ovomucoid inhibitor (Worthington Papain Dissociation Kit) and dissociated cells were passed through a 20 μm cell strainer, spun down at 300 ***g*** and resuspended in 500 μl 0.4 mg/ml BSA (Ambion Ultrapure BSA, AM2616). Propidium iodide (ThermoFisher, P1304MP) was added to the cell suspension (final concentration 50 μg/ml) and cells were sorted using a BD FACS Aria cell sorter at 4°C and collected in 0.4 mg/ml BSA in DNA LowBind tubes (Eppendorf, 0030108523). Cells were pelleted at 300 ***g*** for 5 min at 4°C and manually counted using a Bürker Chamber. After single cell dissociation, single cells were captured and partitioned into droplets using a 10X Chromium controller, and libraries were prepared using Chromium Next GEM Single Cell 3′ Reagent Kits v3.1 according to the manufacturer's instructions. cDNA and post-library quality controls were performed using an Agilent Bioanalyser and DNA concentrations were determined using Qubit dsDNA (ThermoFisher). Libraries were sequenced on an Illuminia NextSeq550 System, using NextSeq 500/550 High Output Kit v2.5, with 150-cycle flow cells (Illumina, 20024907). Library preparations of one sample per genotype were run on a single flow cell, using the following read set-up: read 1, 28 cycles; i7, 8 cycles; i5, not used, single index only; read 2, 130 cycles.

### Single cell RNAseq data processing

FASTQ files were aligned to a reference genome GRCm38 using CellRanger 5.0. CellRanger h5 output files were imported and converted into Seurat Objects using Seurat V4. Quality control was performed for each dataset, excluding cells with the following features: >10% mitochondrial genes, >50,000 RNA counts, <2000 or >10,000 genes or features. After initial quality control, doublets, as indicated by scDbFinder package (version 1.14.0), were removed from each dataset. The expression data were then normalized using SCTransform V2, with regression out of mitochondrial genes. Principal components analysis (PCA) and uniform manifold approximation and projection (UMAP) were used as non-linear dimensionality reduction techniques for cell clustering. Epcam^+^ clusters were identified and subsetted for further analysis. Datasets were integrated using Seurat SCTransform V2 ‘IntegrateData’, and dimensionality reduction settings were determined using ‘ElbowPlot’ and examination of marker gene expression for different clusters. Subsetting of specific clusters (DC and PC) was performed to separate different cell types and these were assigned according to the parental UMAP. A subset of DCs from the wild-type dataset was excluded based on high expression levels of periotic mesenchyme markers *Tbx18* and *Pou4f3*, and was considered to be contamination (for full code availability, see https://github.com/Emma-R-Andersson-Lab). Pseudotime analysis was performed using Monocle (version 1.3.1)

### Immunohistochemistry (whole-mount preparations)

Embryos and pups were collected from timed pregnant females at various embryonic and postnatal timepoints, as indicated. Inner ears were dissected out from the skull and fixed in fresh 4% PFA for 1.5 h at room temperature. Fixed inner ears were washed in PBS overnight at 4°C. Otic cup, Reisner's membrane and the tectorial membrane were subsequently dissected off to expose the sensory epithelium of the cochlea. Cochleae were blocked in PBS containing 10% normal donkey serum (Sigma, D9663) and 0.3% TritonX (Sigma, T8787) for 1 h at room temperature. Cochleae were incubated in primary antibody overnight at 4°C (for primary antibody details, please see Table S10). Cochleae were washed three times for 15 min in PBS and incubated in secondary antibodies in PBS for 1 h at room temperature. After secondary antibody incubation, cochleae were washed three times for 15 min in PBS and the sensory epithelium was dissected and mounted (Sigma-Aldrich, F6182) on a glass slide. Secondary antibodies were used at 1:500, including donkey anti-rabbit Alexa Fluor 488 (Abcam, AB150073), donkey anti-goat Alexa Fluor 546 (ThermoFisher, A11056), donkey anti-goat Alexa Fluor 594 (Abcam, AB150132), donkey anti-sheep Alexa Fluor 555 (Abcam, 150178) and DAPI (Sigma, MBD0015) for nuclear counterstain.

### RNAscope (cryosections)

Embryos and pups were collected from timed pregnant females at various embryonic and postnatal timepoints, as indicated. Inner ears were dissected out from the skull and fixed in fresh 4% PFA overnight in the cold room. Cochleae were exposed to a sucrose gradient (5, 10, 15, 20 and 25% sucrose for 30 min each) and incubated overnight in 30% sucrose at 4°C. Tissues were embedded in OCT medium and stored at −80°C. 12 µm tissue sections were made using a Epredia CryoStar NX70 Cryostat (blade temperature −20°C) NX70 cryostat. RNAscope hybridization was performed according to the manufacturer's instructions (ACDbio). Following RNAscope, sections were incubated with primary antibodies and processed as described in the previous section. For RNAscope probe details, please see Table S10.

### Image acquisition and quantification

Confocal images were acquired using an LSM880 or LSM980 with Zeiss ZEN software. Acquisition parameters were selected to maximize contrast between structures of interest. The sharpest focal plane with the clearest structures was selected for analyses. HCs and SCs were manually counted using ImageJ software. For prosensory domain size measurements, Qupath 0.4.4 or Zeiss Zen software was used using ‘Polygon’ or ‘Draw spline contour’ tool, respectively. For quantification of RNAscope signal, images were acquired using the same imaging settings among samples, and a customized ImageJ script was used by adjusting images to a threshold followed by ‘Measure Particles’. The number of particles was subsequently multiplied by the average particle size and normalized by dividing by the size of the smallest particle per cochlea, as recommended by manufacturer's technical note (TS-46-003).

### Scanning electron microscopy

Cochleae were dissected out and tissue was fixed in fixation buffer (2.5% glutaraldehyde and 2% paraformaldehyde in 0.1 M phosphate buffer) for 1 h at room temperature and stored in buffer at 4°C. After fixation, the sensory epithelium was removed and trimmed for final preparation. Cochleae were dehydrated using a series of ethanol gradients (20-40-60-80-100% ethanol) and were critical point dried using liquid CO_2_. Dried samples were mounted on SEM mini-holders, sputter-coated with platinum and imaged using a Zeiss Gemini Ultra 55 SEM.

### FM1-43 dye staining

Inner ears were dissected out and the otic cup was removed to expose the sensory epithelium. Fresh inner ears were incubated for 60 s in FM1-43 (ThermoFisher, F35355) in PBS (final concentration 5 µM), washed with 1×PBS and kept in the dark. After washing, cochleae were fixed in 4% PFA and processes as described in the immunohistochemistry section.

## Supplementary Material



10.1242/develop.202949_sup1 Supplementary information

Table S3. Marker genes for individual cell types for all Epcam^+^ cell populations (*Jag1^Ndr/Ndr^* and *Jag1^+/+^* combined).

Table S4. Pseudo bulk analysis identified differently expressed genes for *Jag1^Ndr/Ndr^* versus *Jag1^+/+^*, pathway enrichment for differently expressed genes and previously reported Jag1 mediated genes.

Table S5. Differentially expressed genes per cell type for *Jag1^Ndr/Ndr^* versus *Jag1^+/+^*

Table S6. Pathway enrichment for differently expressed genes per cell type for *Jag1^Ndr/Ndr^* versus *Jag1^+/+^*

Table S7. Marker genes for individual cell types for all Epcam+ cell populations for *Jag1^+/+^* dataset

Table S8. Number of cells per cell type per genotype

Table S9. OHC sub clustering analysis and PC signature removal
