# Supplementary material for: Jag1 represses Notch activation in lateral supporting cells and inhibits an outer hair cell fate in the medial cochlea
Source: Development. 2024 Nov 5;151(21):dev202949. doi: 10.1242/dev.202949 (PMC11574350; doi:10.1242/dev.202949)
Supplement: Supplementary information [file develop-151-202949-s1.pdf]

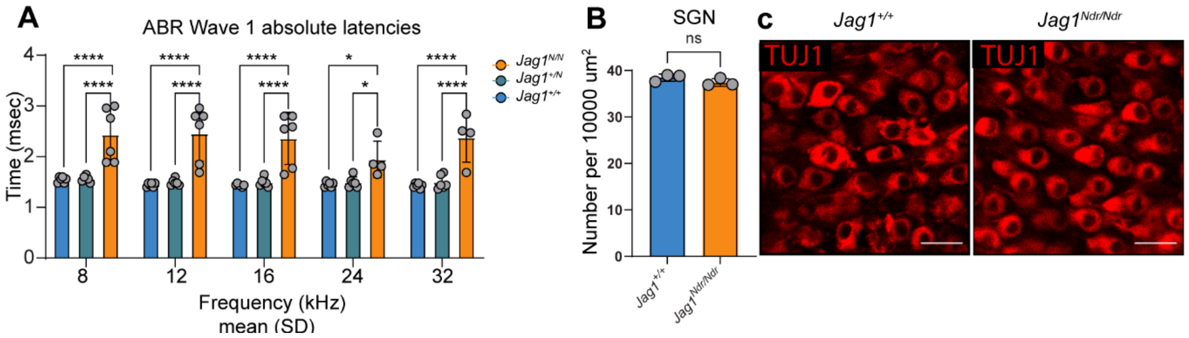

Fig S1. *Jag1*$^{Ndr/Ndr}$ mice display delayed auditory response in absence of spiral ganglion nuclei reduction. (A) ABR wave-1 absolute latencies, indicating a delay in auditory response along all frequencies, n=6 per genotype. (B) Quantification of the number of spiral ganglion nuclei (SGN) per 1000 µm2, indicating no difference in SGN abundance between *Jag1*$^{Ndr/Ndr}$ and *Jag1*$^{+/+}$ mice, P50, n=3 per genotype with 15 sections quantified per animal. (C) Confocal image showing SGN nuclei at P50 (Tuj1). Scale bar represents 20 µm. One-way ANOVA with Bonferroni correction for (A), unpaired t-test for data displayed in (B).

Development • Supplementary information

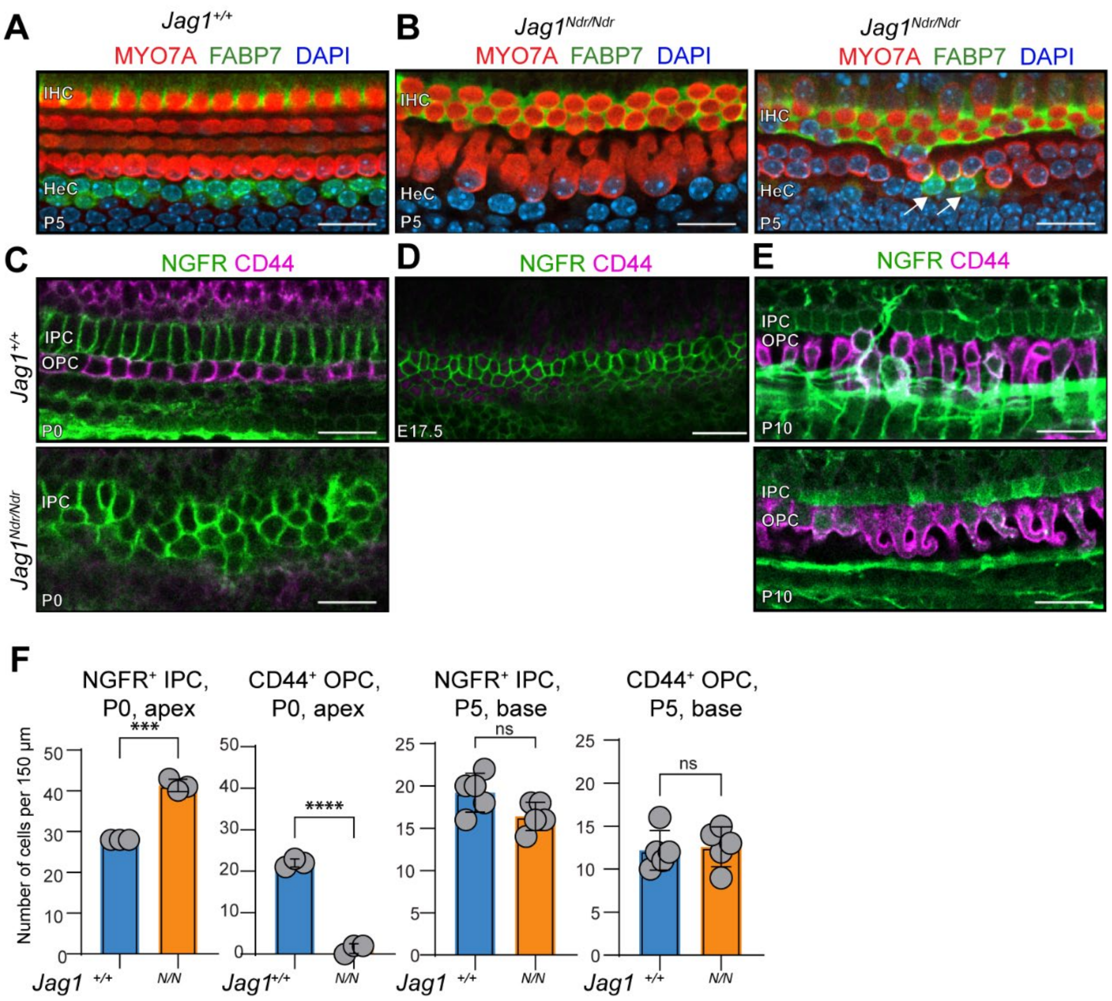

Fig S2. *Jag1$^{Ndr/Ndr}$* mice show sporadic remnant HeCs and have delayed PC development. (A) *Jag1$^{+/+}$* HeC phenotype showing HeCs (FABP7, green, also labels IPhCs), HCs (MYO7A, red) and nuclei (DAPI, blue) showing FABP7-positive cell rows in the IHC/IPhC and HeC positions. (B) Representative image (left) of *Jag1$^{Ndr/Ndr}$* HeC phenotype stained for HeCs (FABP7, green), HCs (MYO7A, red) and nuclei (DAPI, blue) showing the absence of FABP7-positive cell layers lateral to OHCs, in the HeC position, however, sporadic FABP7^{+} HeCs can be found (right). (C) PC phenotype at P0, showing IPCs (NGFR, green) and OPCs (CD44, magenta). (D) Pillar cell phenotype at E17.5 in *Jag1$^{+/+}$* animals demonstrating widespread NGFR signal. (E) At P10, the number of IPCs and OPCs is similar in *Jag1$^{Ndr/Ndr}$* and *Jag1$^{+/+}$* mice (F) Quantification of PCs for P0 apical region, indicating an increase in NGFR positive cells, and a reduction of CD44 positive cells 40.2± 3.1 NGFR+ cells per 150 µm in *Jag1$^{Ndr/Ndr}$* compared to 29.2±1.8 NGFR+ cells per 150 µm in *Jag1$^{+/+}$*, p-value <0.001, mean ± SD and 1.2±1.1 CD44+ cells per 150 µm in *Jag1$^{Ndr/Ndr}$* compared to 23.8±2.8 CD44+ cells per 150 µm in *Jag1$^{+/+}$*, p-value <0.001, mean ± SD. At P5, base region, the number of IPC and OPCs is similar for *Jag1$^{Ndr/Ndr}$* and *Jag1$^{+/+}$* mice 16.4±1.7 IPC cells per 150 µm in *Jag1$^{Ndr/Ndr}$* compared to 19.2±2.3 in *Jag1$^{+/+}$*, p-value =ns and 12.6±2.3 OPC cells per 150 µm in *Jag1$^{Ndr/Ndr}$* compared to 12.2±2.3 in *Jag1$^{+/+}$*, p-value =ns, mean ± SD. n=5 per genotype, with exception of P10 PC data (n=2); data are mean with standard deviation, scale bar represents 20 um; *p-value <0.05; **p-value <0.01; ***p-value <0.001, ****p-value <0.0001, unpaired t-test.

Development • Supplementary information

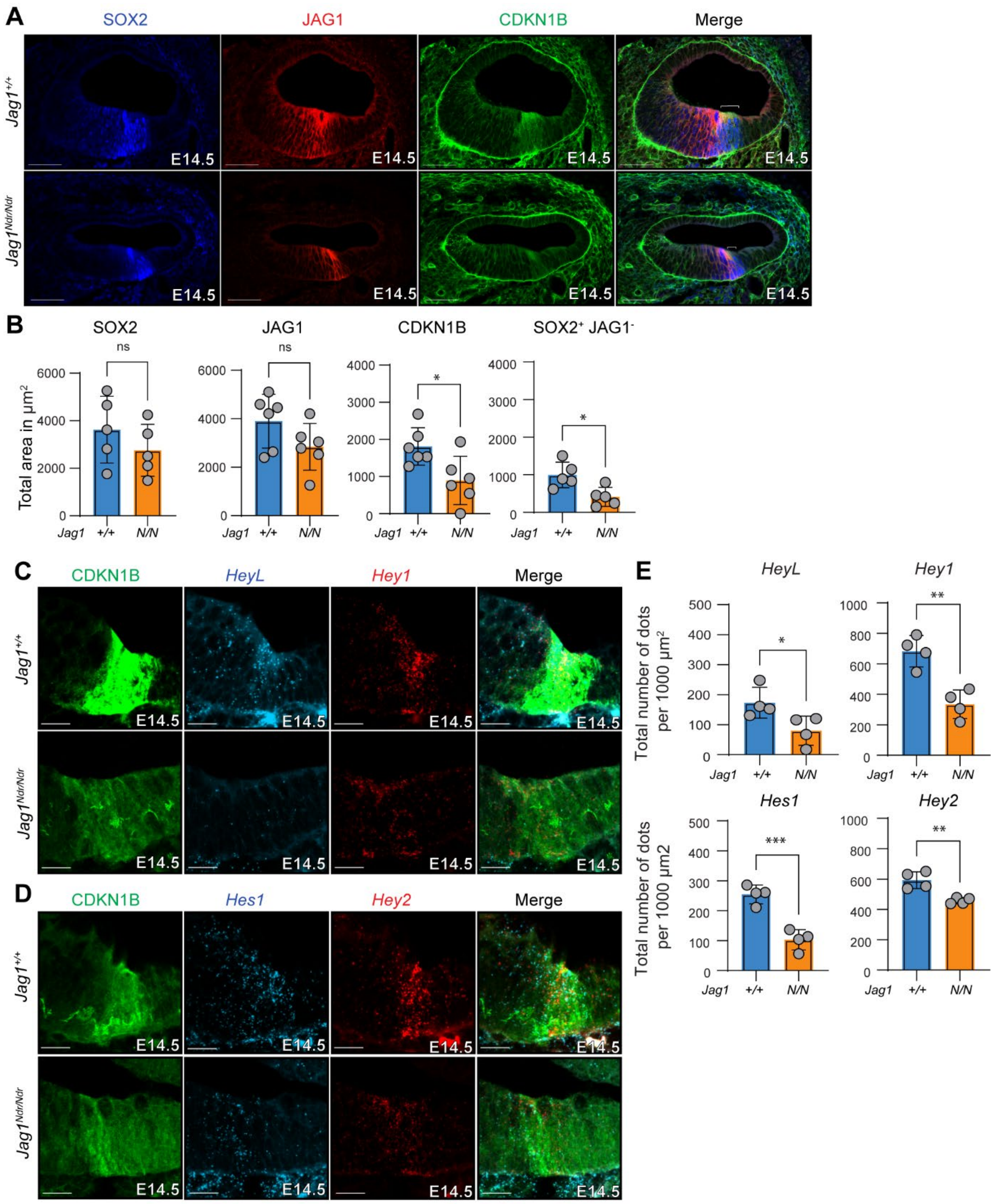

Fig S3. The E14.5 *Jag1*$^{Ndr/Ndr}$ prosensory domain is smaller and exhibits reduced Notch activation. (A-B) Prosensory domain marker expression in the basal turn, including SOX2 (blue), JAG1 (red) and CDKN1B (green) for *Jag1*$^{+/+}$ mice (upper panels) and *Jag1*$^{Ndr/Ndr}$ mice (lower panels), indicating a smaller CDKN1B domain as well as a reduction in the SOX2-positive/JAG1-negative lateral prosensory domain (brackets), quantified in (B). (C-D) Notch target gene mRNA expression in the basal turn, showing reduced *HeyL, Hey1, Hes1* and *Hey2* expression in *Jag1*$^{Ndr/Ndr}$ mice, quantified in (E) Number of dots for *Heyl* 80.0 ±48.4 in *Jag1*$^{Ndr/Ndr}$ compared to 173.3± 51.4 in *Jag1*$^{+/+}$ p-value <0.001, *Hey1* 334.5± 93.7 in *Jag1*$^{Ndr/Ndr}$ compared to 684.0± 103.1 in *Jag1*$^{+/+}$ p-value <0.01, *Hey2* 457.3 ±93.7 in *Jag1*$^{Ndr/Ndr}$ compared to 592.8± 55.5 in *Jag1*$^{+/+}$ p-value <0.01, *Hes1* in *Jag1*$^{Ndr/Ndr}$ 102.5± 33.4 compared to 254.3± 31.1 in *Jag1*$^{+/+}$, p-value <0.001 n=5 per genotype; data is mean with standard deviation; scale bar represents 20 um; *p-value <0.05; **p-value <0.01; ***p-value <0.001, ****p-value <0.0001, unpaired t-test.

Development • Supplementary information

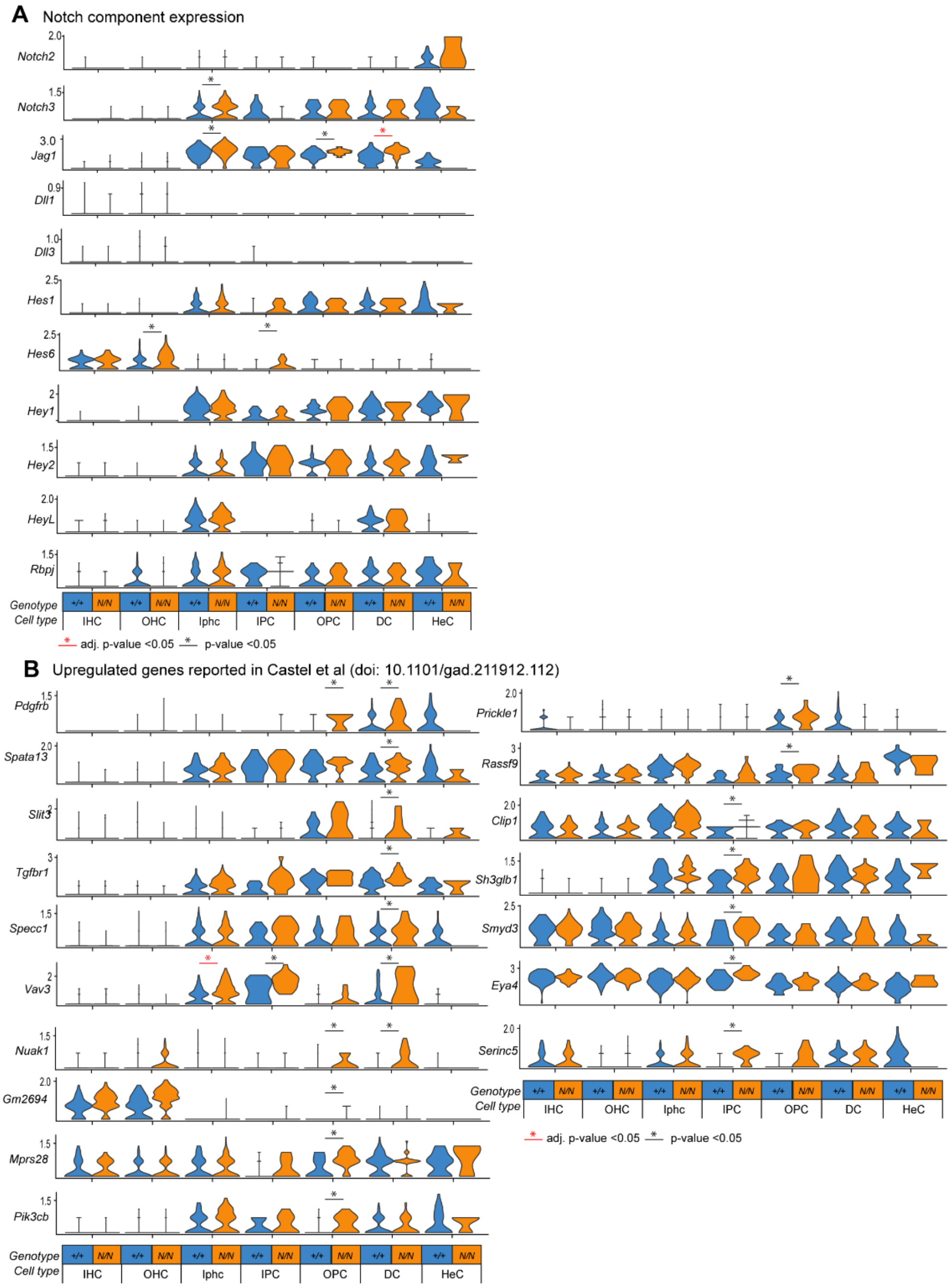

Fig S4. mRNA expression of Notch components and genes with inducible Rbpj binding sites. (A) Violin plots showing the expression of Notch components in different cell types of the Organ of Corti, split per genotype. (B) Dysregulation of genes with an inducible Rbpj binding sites[41], in lateral *Jag1*$^{Ndr/Ndr}$ SCs. Red asterisks indicate significant upregulation (adj. p-value <0.05); black asterisks indicate potential upregulation (p-value <0.05).

Development • Supplementary information

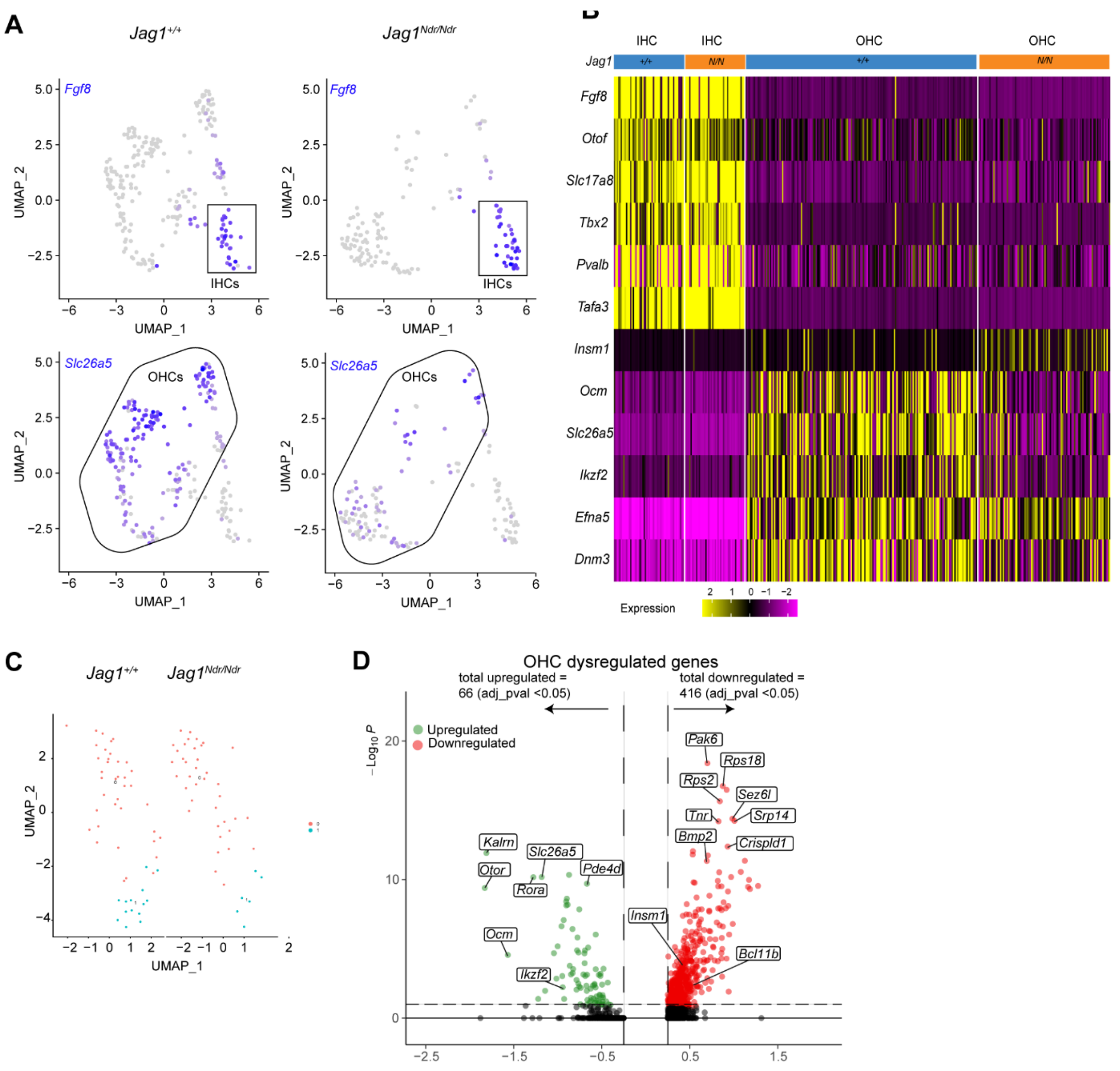

Fig S5. Subclustering and renormalization of HC populations. (A) UMAP projection of subsetted and renormalized HC population, split by genotype. (B) Volcano plot showing downregulated genes (left, green) and upregulated genes (right, red) in *Jag1*$^{Ndr/Ndr}$ OHCs compared to *Jag*$^{+/+}$ OHCs, for the dataset shown in (A). (C) Pseudotime analysis of OHC population shown in (A), demonstrating that there is no significant difference in OHC pseudotime for *Jag1*$^{Ndr/Ndr}$ OHCs compared to *Jag*$^{+/+}$ OHCs. (D) Volcano plot showing up and down regulated genes for OHCs after subsetting. Data are mean with standard deviation; unpaired t-test.

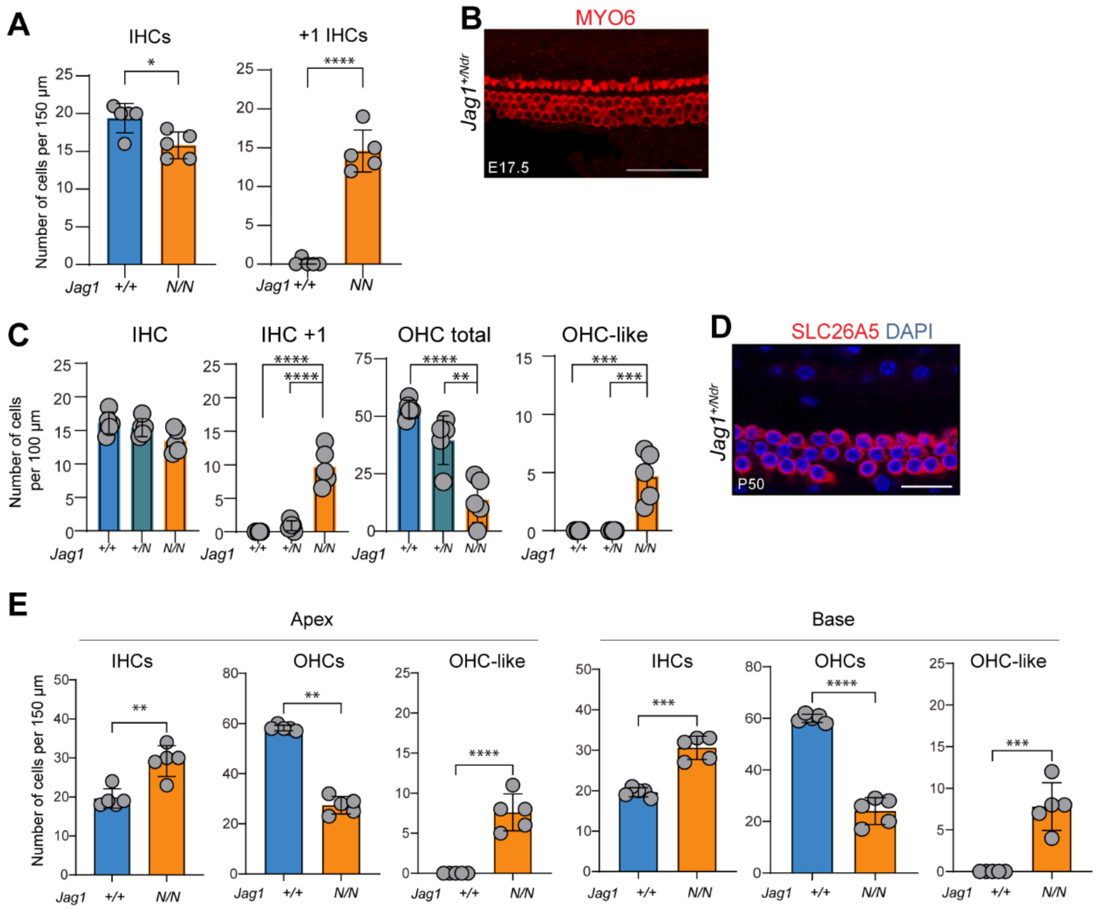

Fig S6. OHC-like cells are not present in *Jag1*$^{+/Ndr}$ mice. (A) E17.5 quantification of IHCs and supernumerary IHCs (IHC +1). (B) HC phenotype of *Jag1*$^{+/Ndr}$ mice at E17.5 showing occasional supernumerary IHCs, but no OHC-like cells (MYO6, red). Scalebar = 50 µm. (C) Quantification of various HC subtypes for all genotypes at 17.5. (D) *Jag1*$^{+/Ndr}$ phenotype at P50 showing OHC marker SLC26A5 (red) expression in the lateral OHC domain, but not outside of the lateral domain, indicating that there are no OHC-like cells in *Jag1*$^{+/Ndr}$ mice. Scalebar = 50 µm. n=5 per genotype; data are mean with standard deviation; *p-value <0.05; **p-value <0.01; ***p-value <0.001, ****p-value <0.0001.

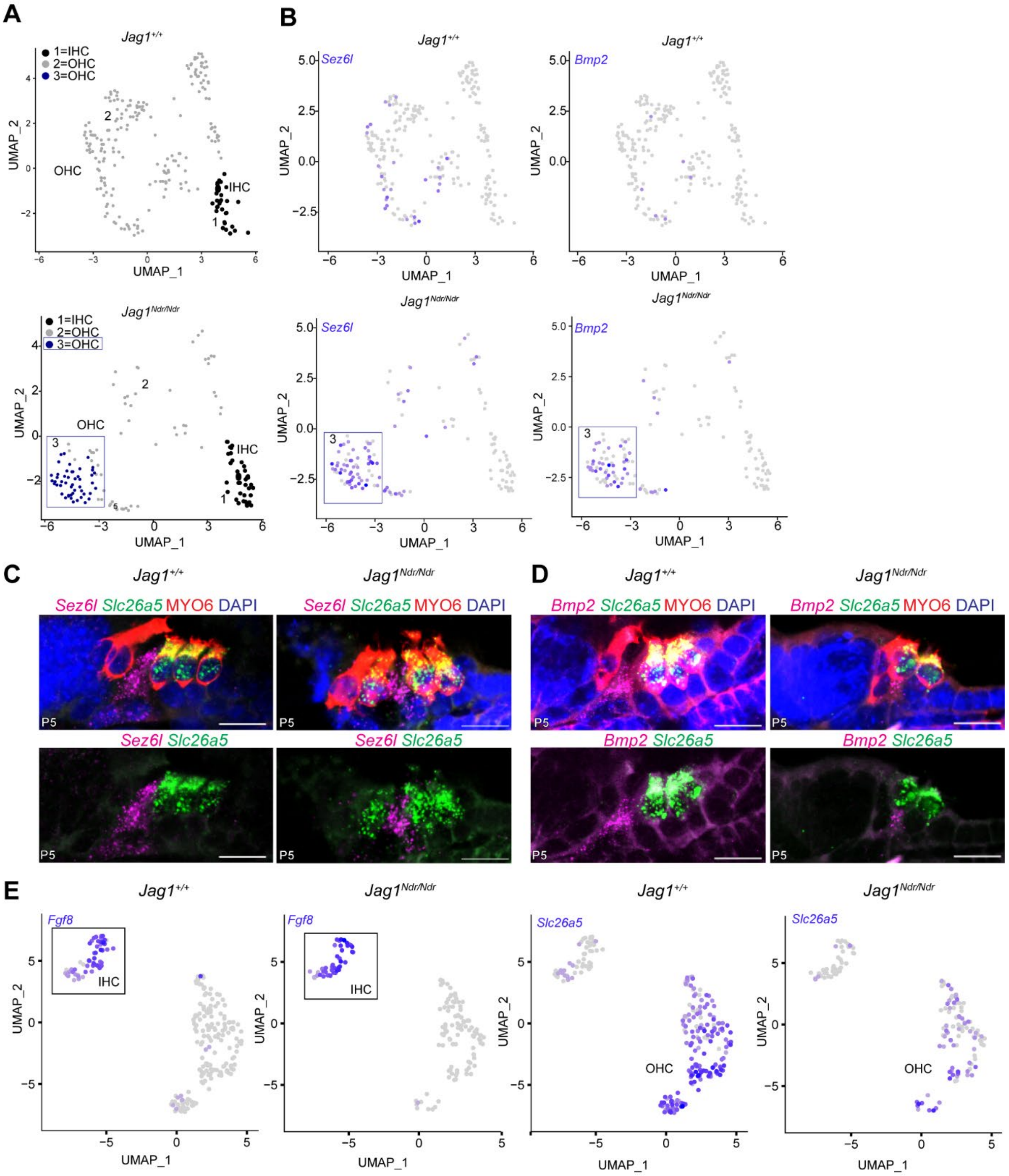

Fig. S7. *Sez6l* and *Bmp2* expression in *Jag1*$^{Ndr/Ndr}$ OHC datasets and mRNA validation. (A) UMAP projection of unsupervised clustering of the renormalized HC subset, indicating an OHC population exclusively found in *Jag1*$^{Ndr/Ndr}$ OHCs (lower panel, cluster3, blue). (B) Featureplot showing expression of *Sez6l* and *Bmp2*, indicating high expression of Sez6l and Bmp2 in cluster 3. (C-D) RNAscope for mRNA expression of *Sez6l* and *Bmp2* indicates expression of *Sez6l* and *Bmp2* in PCs in both *Jag1*$^{Ndr/Ndr}$ and *Jag*$^{+/+}$ mice, and absence of *Sez6l* and *Bmp2* in lOHC-like cells (C) and bOHC-like cells (D). (E) UMAP projection of OHCs subset with removal of a PC signature, showing similar clusters and lack of separation of additional clusters in *Jag1*$^{Ndr/Ndr}$ population, formerly present in (A). n=3 for phenotypic mRNA analysis, with 15 sections analysed per animal.

Development • Supplementary information

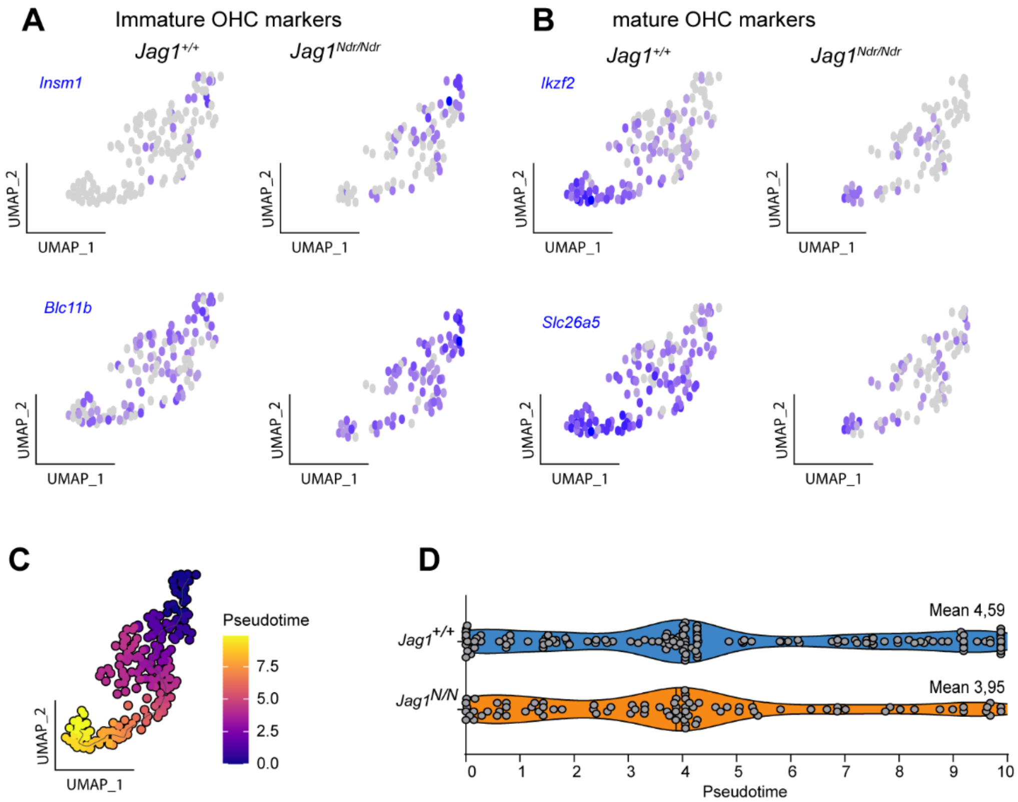

Fig. S8. Expression of immature and mature marker genes in OHC subset and pseudotime analysis. (A) UMAP showing expression of immature markers *Insm1* and *Blc11b* in OHC subset. (B) UMAP showing expression of mature markers *Ikzf2* and *Slc26a5* in OHC subset. (C) UMAP showing pseudotime. (D) Pseudotime analysis of OHC population shown in (A), demonstrating that there is no significant difference in OHC pseudotime for *Jag1*$^{Ndr/Ndr}$ OHCs compared to *Jag*$^{+/+}$ OHCs (4.0±2.9 in *Jag1*$^{Ndr/Ndr}$ compared to 4.6±3.1 in *Jag1*$^{+/+}$, p-value =ns, mean ±SD).

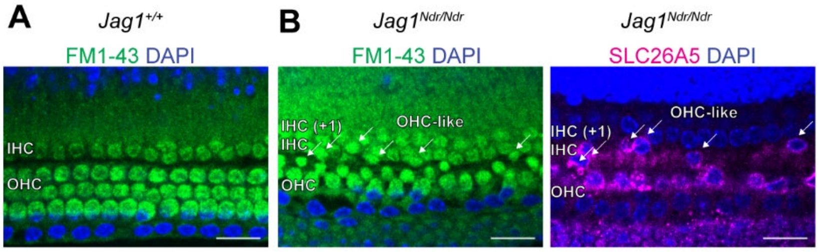

Fig S9. OHC-like cells do take up HC dye FM1-43. (A) FM1-43 uptake in *Jag*$^{+/+}$ HCs (green), indicating that IHCs and OHCs take up FM1-43 dye. (B) FM1-43 uptake in *Jag1*$^{Ndr/Ndr}$ HCs (green), indicating that all HCs take up FM1-43 dye (left), including OHC-like cells (right, arrows). Scale bar represents 20um.

Development • Supplementary information

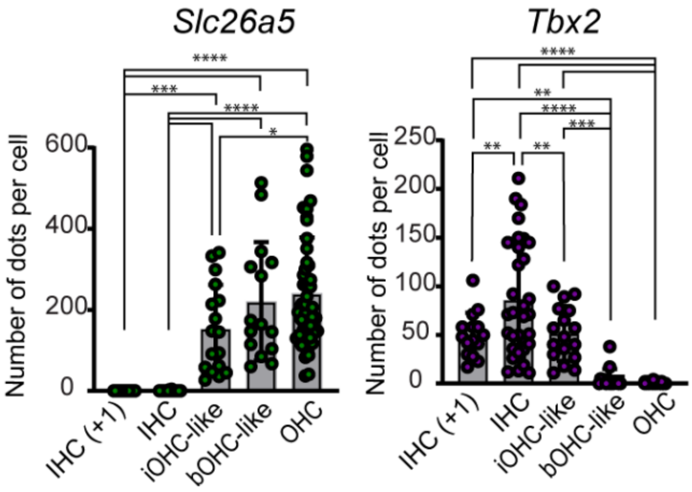

Fig. S10. iOHC-like cell are Tbx2 positive. Quantification of *Slc26a5* (left) and *Tbx2* (right) puncta in *Jag1*$^{Ndr/Ndr}$ HC subtypes, 0.0±0.0 *Slc26a5* dots per IHC, 0.6±1.1 dots per +1 IHC, 153.2±108.3 dots per iOHC-like cell, 220.7±146.3 dots per bOHC-like cell and 240.4±138.2 dots per OHC, mean ± SD (B) Quantification of *Tbx2* mRNA puncta (right) in *Jag1*$^{Ndr/Ndr}$ HC subtypes, 85±58 dots per IHC, 50±22 dots per +1 IHC, 51±28 dots per iOHC-like cell, 4±10 dots per bOHC-like cell and 0.47±0.94 dots per OHC, mean ± SD, data are mean with standard deviation. *p-value <0.05; **p-value <0.01; ***p-value <0.001, ****p-value <0.0001, one-way ANOVA with Bonferroni correction. n=3 animals per genotype, each dot is one cell. These data correspond to the animals shown in Fig 5C,E.

Table S1. Summary of Notch defective animal models reporting an inner ear phenotype, extended table of Brown et al Table1[1]

Receptors	Type of mutation	Phenotype	Ref
Notch1	Inner ear specific knockout (Pax2-cre)	Extra OHC row, correctly patterned	2
Notch1	Inner ear specific knockout (Foxg1-cre)	3-fold increase in IHCs and OHCs, disorganized	3
Notch1	SC specific knockout (Sox2CreER x Notch1fl/fl) at P0/P1	Loss of lateral SCs, and HC loss secondary to SCs loss.	4
**Ligands**	**Type of mutation**	**Phenotype**	**Ref**
Jag1	Headturner ($Htu^{+/-}$) ENU-induced missense mutation (G289D)	Ectopic IHCs, reduction OHCs	5
Jag1	Ozzy ($Ozzy^{+/-}$) ENU-induced missense mutation (W167R)	Ectopic IHCs, reduction OHCs	6
Jag1	Slalom ($Slm^{+/-}$) ENU-induced missense mutation (P269S)	Ectopic IHCs, reduction OHCs	7
Jag1	*Inner ear specific knockout (Foxg1-cre)* from E8.75	Duplication of IHCs, lack of OHCs in mid cochlea	8
Jag1	SC specific knockout ($Sox2^{CreERT2}/Jag1^{loxP/loxP}$)	Loss of HeC	9
	Inner ear specific knockout ($Fgfr3^{iCreERT2}/Jag1^{loxP/loxP}$)	Loss of HeC	9
Jag1	SC specific knockout ($Sox2^{CreER}/Jag1^{fl/fl}$)	Loss of some HeC	4
Jag2	Null mutant, heterozygous ($Jag2^{+/\square DSL}$)	Ectopic IHCs	10
Jag2	Null mutant, homozygous $Jag2^{DSL/DSL}$	Duplication of IHCs, extra row of OHC, abnormal orientation of stereocilia on OHCs.	11
Dll1	Null mutant, heterozygous ($DLL1^{Dll1ki/+}$)	Ectopic IHCs and supernumerary OHCs	12
Dll1	Null mutant, homozygous ($DLL1^{Dll1ki/LacZ}$)	Ectopic IHCs and supernumerary OHCs	12
Dll1	Inner ear specific knockout *(Foxg1-cre)*	Ectopic IHCs, supernumerary OHCs, and delayed cochlear growth	13
Dll1+ Jag2	Double null mutant, heterozygous ($Jag2^{+/\square DSL}$ / $DLL1^{+/LacZ}$)	Duplication of IHCs, extra row of OHCs	12
Dll1+ Jag2	Double null mutant, ($Jag2^{\square\square DSL/\square DSL}$ / $DLL1^{+/LacZ}$)	Duplication of IHCs, extra row of OHCs, disorganised	12
Dll1+ Jag2	Double null mutant, ($Jag2^{\square\square DSL/\square DSL}$ / $DLL1^{+/Dll1ki}$)	Two to four rows of IHCs, four to six rows of OHCs, disorganised	12

Development • Supplementary information

Dll3	Null mutant ($DLL3^{pu/pu}$)	No phenotype	14
**Modulators**	**Type of mutation**	**Phenotype**	**Ref**
Lfng	Null mutant ($Lfng^{LacZ/LacZ}$)	No phenotype	2
Mfng	Null mutant ($Mfng^{Tm1.1Cfg/tm1.1Cfg}$)	No phenotype	15
Lfng + Mfng	Double null mutant ($Lfng^{LacZ/LacZ}$/ $Mfng^{Tm1.1Cfg/tm1.1Cfg}$)	Ectopic IHCs and Iphcs	15
Lfng + Jag2	Double null mutant $Jag2^{\Delta DSL/\Delta DSL}$ / $Lfng^{lacZ/lacZ}$	Supernumerary OHCs, and abnormal stereocilia on OHCs. Rescues Jag2 IHC phenotype	16
Pofut1	Null mutant ($Pofut1^{-/-}$)	Ectopic IHCs	15
Pofut1	Inner ear specific knockout (Pax2-cre)	Ectopic IHCs and Iphcs, supernumerary OHCs	17
**Downstream targets**	**Type of mutation**	**Phenotype**	**Ref**
Hes1	Null mutant, heterozygous ($Hes1^{+/-}$)	Ectopic IHCs	18
Hes1	Null mutant, homozygous ($Hes1^{-/-}$)	Duplication IHCs	18
Hes5	Null mutatant, homozygous ($Hes5^{-/-}$) (null mutation)	Supernumerary OHCs	18
Hes5 + Hes1	Double null mutant ($Hes1^{-/-}$ / $Hes5^{+/-}$)	Duplication of IHCs	18
Hes5 + Hes1	Double null mutant ($Hes1^{+/-}$ / $Hes5^{-/-}$)	Ectopic IHCs and supernumerary OHCs	18
Hey2	Null mutant, homozygous ($Hey2^{-/-}$)	Supernumerary OHCs	19
Hey2 + Hes5	Double null mutant, homozygous ($Hey2^{-/-}$/ $Hes5^{-/-}$)	Supernumerary OHCs	19
Hey2 + Hes5	Double null mutant, homozygous ($Hey2^{-/-}$/ $Hes5^{-/-}$)	Supernumerary OHCs	19
Hey2	Null mutant, homozygous ($Hey2^{-/-}$)	No phenotype, but additional DAPT treatment results in loss of PC and DCs.	20
Hey2	Null mutant, homozygous ($Hey2^{-/-}$)	No phenotype	21
Hey2 + Hes1	Double null mutant ($Hey2^{+/-}$/ $Hes1^{-/-}$)	Ectopic IHCs and supernumerary OHCs	19
Hey1	Floxed knockout ($Hey1^{fl/fl}$)	No phenotype	21

Development • Supplementary information

Hey1 + Hey2	Double null mutant *Hey1*$^{-/-}$/ *Hey1*$^{-/-}$	Ectopic HCs in the PC region	21
Co-activators	**Type of mutation**	**Phenotype**	**Ref**
dnMaml	Inner ear specific knockout (Pax2-cre)	Ectopic IHCs and Iphcs	15
dnMaml	Inner ear specific knockout (Pax2-cre)	Loss of DCs	22
Rbpj	Inner ear specific knockout (Foxg1-cre)	Mice die before before pattern establishment	23
Rbpj	Inner ear specific knockout (Pax2-cre)	Mice die before before pattern establishment	24
Rbpj	Inner ear specific knockout *(Fgfr3-iCreER; Rbpj*$^{-/\Delta}$)	Loss of DCs	22

References

1. Brown, R. & Groves, A. K. Hear, hear for notch: Control of cell fates in the inner ear by notch signaling. *Biomolecules* vol. 10 Preprint at https://doi.org/10.3390/biom10030370 (2020).
2. Zhang, N., Martin, G. V., Kelley, M. W. & Gridley, T. A mutation in the Lunatic fringe gene suppresses the effects of a Jagged2 mutation on inner hair cell development in the cochlea. *Current Biology* **10**, 659–662 (2000).
3. Kiernan, A. E., Cordes, R., Kopan, R., Gossler, A. & Gridley, T. The Notch ligands DLL1 and JAG2 act synergistically to regulate hair cell development in the mammalian inner ear. *Development* **132**, 4353–62 (2005).
4. Heffer, A., Gilels, F. A. & Kiernan, A. E. Deletion of Notch1 during Cochlear Maturation Leads to Rapid Supporting Cell Death and Profound Deafness. *Journal of Neuroscience* **43**, (2023).
5. Kiernan, A. E. *et al.* The Notch ligand Jagged1 is required for inner ear sensory development. *Proc Natl Acad Sci U S A* **98**, 3873–8 (2001).
6. Vrijens, K. *et al.* Ozzy, a Jag1 vestibular mouse mutant, displays characteristics of Alagille syndrome. *Neurobiol Dis* **24**, 28–40 (2006).
7. Tsai, H. *et al.* The mouse slalom mutant demonstrates a role for Jagged1 in neuroepithelial patterning in the organ of Corti. *Hum Mol Genet* **10**, 507–512 (2001).
8. Brooker, R., Hozumi, K. & Lewis, J. Notch ligands with contrasting functions: Jagged1 and Delta1 in the mouse inner ear. *Development* **133**, 1277–1286 (2006).
9. Chrysostomou, E. *et al.* The notch ligand jagged1 is required for the formation, maintenance, and survival of Hensen's cells in the mouse cochlea. *Journal of Neuroscience* **40**, (2020).
10. Lanford, P. J. *et al.* Notch signalling pathway mediates hair cell development in mammalian cochlea. *Nat Genet* (1999) doi:10.1038/6804.
11. Lanford, P. J. *et al.* Notch signalling pathway mediates hair cell development in mammalian cochlea. *Nat Genet* **21**, 289–292 (1999).
12. Kiernan, A. E., Cordes, R., Kopan, R., Gossler, A. & Gridley, T. The Notch ligands DLL1 and JAG2 act synergistically to regulate hair cell development in the mammalian inner ear. *Development* **132**, 4353–62 (2005).
13. Brooker, R., Hozumi, K. & Lewis, J. Notch ligands with contrasting functions: Jagged1 and Delta1 in the mouse inner ear. *Development* **133**, 1277–1286 (2006).
14. Hartman, B. H., Hayashi, T., Nelson, B. R., Bermingham-McDonogh, O. & Reh, T. A. Dll3 is expressed in developing hair cells in the mammalian cochlea. *Developmental Dynamics* (2007) doi:10.1002/dvdy.21307.
15. Basch, M. L. *et al.* Fine-tuning of Notch signaling sets the boundary of the organ of Corti and establishes sensory cell fates. *Elife* **5**, 841–850 (2016).

Development • Supplementary information

16. Zhang, N., Martin, G. V., Kelley, M. W. & Gridley, T. A mutation in the Lunatic fringe gene suppresses the effects of a Jagged2 mutation on inner hair cell development in the cochlea. *Current Biology* **10**, 659–662 (2000).
17. Chen, P., Johnson, J. E., Zoghbi, H. Y. & Segil, N. The role of Math1 in inner ear development: Uncoupling the establishment of the sensory primordium from hair cell fate determination. *Development* **129**, (2002).
18. Zine, A. *et al.* Hes1 and Hes5 activities are required for the normal development of the hair cells in the mammalian inner ear. *J Neurosci* **21**, 4712–20 (2001).
19. Li, S. *et al.* Hey2 functions in parallel with Hes1 and Hes5 for mammalian auditory sensory organ development. *BMC Dev Biol* (2008) doi:10.1186/1471-213X-8-20.
20. Doetzlhofer, A. *et al.* Hey2 Regulation by FGF Provides a Notch-Independent Mechanism for Maintaining Pillar Cell Fate in the Organ of Corti. *Dev Cell* (2009) doi:10.1016/j.devcel.2008.11.008.
21. Benito-Gonzalez, A. & Doetzlhofer, A. Hey1 and Hey2 control the spatial and temporal pattern of mammalian auditory hair cell differentiation downstream of hedgehog signaling. *Journal of Neuroscience* **34**, (2014).
22. Campbell, D. P., Chrysostomou, E. & Doetzlhofer, A. Canonical Notch signaling plays an instructive role in auditory supporting cell development. *Sci Rep* **6**, 19484 (2016).
23. Yamamoto, N., Chang, W. & Kelley, M. W. Rbpj regulates development of prosensory cells in the mammalian inner ear. *Dev Biol* **353**, 367–379 (2011).
24. Basch, M. L., Hyama, T., Segil, N. & Groves, A. K. Canonical Notch Signaling Is Not Necessary for Prosensory Induction in the Mouse Cochlea: Insights from a Conditional Mutant of RBPjκ. *Journal of Neuroscience* **31**, (2011).

Development • Supplementary information

Table S2. Summary of hearing function and middle ear bone morphology in *Jag1* mutant mouse models.

Model	Hearing function	Middle ear bones	Ref
Conditional knockout *(Wnt1-Cre; $Jag1^{f/f}$)*	Elevated hearing thresholds among all frequencies	Malformed (columnar) stapes and occasional ectopic processes on incus	[1]
Headturner (*$Htu^{+/-}$*) ENU-induced missense mutation (G289D)	Elevated hearing thresholds, not significant	Not assessed	[2]
Ozzy (*$Ozzy^{+/-}$*) ENU-induced missense mutation (W167R)	Slightly elevated hearing thresholds, most pronounced at middle frequencies	Not assessed	[3]
Slalom (*$Slm^{+/-}$*) ENU-induced missense mutation (P269S)	Not assessed	Not assessed	[4]
Inner ear specific knockout (Foxg1-cre) from E8.75	Not assessed	Not assessed	[5]
SC specific knockout (*$Sox2^{CreERT2}$/$Jag1^{loxP/loxP}$*)	Not assessed	Not assessed	[6]
Inner ear specific knockout (*$Fgfr3^{iCreERT2}$/$Jag1^{loxP/loxP}$*)	Elevated thresholds, deafness at lower frequency range	Not assessed	[6]
SC specific knockout (*$Sox2^{CreER}$/$Jag1^{fl/fl}$*)	Deaf at all frequencies	Not assessed	[7]

1. Teng, C. S. et al. Requirement for Jagged1-Notch2 signaling in patterning the bones of the mouse and human middle ear. Sci Rep 7, 2497 (2017).
2. Kiernan, A. E. et al. The Notch ligand Jagged1 is required for inner ear sensory development. Proc Natl Acad Sci U S A 98, 3873–8 (2001).
3. Vrijens, K. et al. Ozzy, a Jag1 vestibular mouse mutant, displays characteristics of Alagille syndrome. Neurobiol Dis 24, 28–40 (2006).
4. Tsai, H. et al. The mouse slalom mutant demonstrates a role for Jagged1 in neuroepithelial patterning in the organ of Corti. Hum Mol Genet 10, 507–512 (2001).
5. Brooker, R., Hozumi, K. & Lewis, J. Notch ligands with contrasting functions: Jagged1 and Delta1 in the mouse inner ear. Development 133, 1277–1286 (2006).
6. Chrysostomou, E. et al. The notch ligand jagged1 is required for the formation, maintenance, and survival of Hensen's cells in the mouse cochlea. Journal of Neuroscience 40, (2020).
7. Gilels, F. A., Wang, J., Bullen, A., White, P. M. & Kiernan, A. E. Deletion of the Notch ligand Jagged1 during cochlear maturation leads to inner hair cell defects and hearing loss. Cell Death Dis 13, (2022).

Table S3. Marker genes for individual cell types for all Epcam$^+$ cell populations (*Jag1*$^{Ndr/Ndr}$ and *Jag1*$^{+/+}$ combined).

Available for download at
https://journals.biologists.com/dev/article-lookup/doi/10.1242/dev.202949#supplementary-data

Table S4. Pseudo bulk analysis identified differently expressed genes for *Jag1*$^{Ndr/Ndr}$ versus *Jag1*$^{+/+}$, pathway enrichment for differently expressed genes and previously reported Jag1 mediated genes.

Available for download at
https://journals.biologists.com/dev/article-lookup/doi/10.1242/dev.202949#supplementary-data

Table S5. Differentially expressed genes per cell type for *Jag1*$^{Ndr/Ndr}$ versus *Jag1*$^{+/+}$

Available for download at
https://journals.biologists.com/dev/article-lookup/doi/10.1242/dev.202949#supplementary-data

Table S6. Pathway enrichment for differently expressed genes per cell type for *Jag1*$^{Ndr/Ndr}$ versus *Jag1*$^{+/+}$

Available for download at
https://journals.biologists.com/dev/article-lookup/doi/10.1242/dev.202949#supplementary-data

Table S7. Marker genes for individual cell types for all Epcam$^+$ cell populations for *Jag1*$^{+/+}$ dataset

Available for download at
https://journals.biologists.com/dev/article-lookup/doi/10.1242/dev.202949#supplementary-data

Table S8. Number of cells per cell type per genotype

Available for download at
https://journals.biologists.com/dev/article-lookup/doi/10.1242/dev.202949#supplementary-data

Table S9. OHC sub clustering analysis and PC signature removal

Available for download at
https://journals.biologists.com/dev/article-lookup/doi/10.1242/dev.202949#supplementary-data

Table S10. Primary antibodies and RNAscope probes

Primary antibodies

Antibody	Vendor	Cat. Number	Dilution
Primary antibodies			
Cd44	BD Biosciences	550538	1:500
E-cadherin	BD Biosciences	610182	1:500
FABP7	R&D	AF3166	1:1000
Jag1	Cell. Sig.28H8	2620	1:125
Myosin6	BioProteus	25-679	1:1000
NGFR	R&D	AF367	1:500
Ocomodulin	Novus Biologicals	NBP2-14568	1:500
P27kip1	BD Biosciences	610242	1:250
PCMA2	Invitrogen	PA1-915	1:1000
Phalloidin	ThermoFisher	A22287	1:500
Slc26a5	Abcam	Ab242128	1:20.000
Sox2	R&D	AF2018	1:250
Vglut3	Millipore	AB5421-I	1:1000
Tuij1	Biolegend	801209	1:500
RFP	Rockland	200-101-379	1:500
GFP	Rockland	600-401-379	1:500
RNA scope probes			
Hes1	ACDbio	417701-C1	-
Hey1	ACDbio	319021-C3	1:50
Hey2	ACDbio	404651-C2	1:50
HeyL	ACDbio	446881-C1	-
Slc26a5	ACDbio	521321-C1	-
Sez6l	ACDbio	492631-C3	1:50
Bmp2	ACDbio	406661-C2	1:50
Tbx2	ACDbio	448991-C2	1:50

Development • Supplementary information

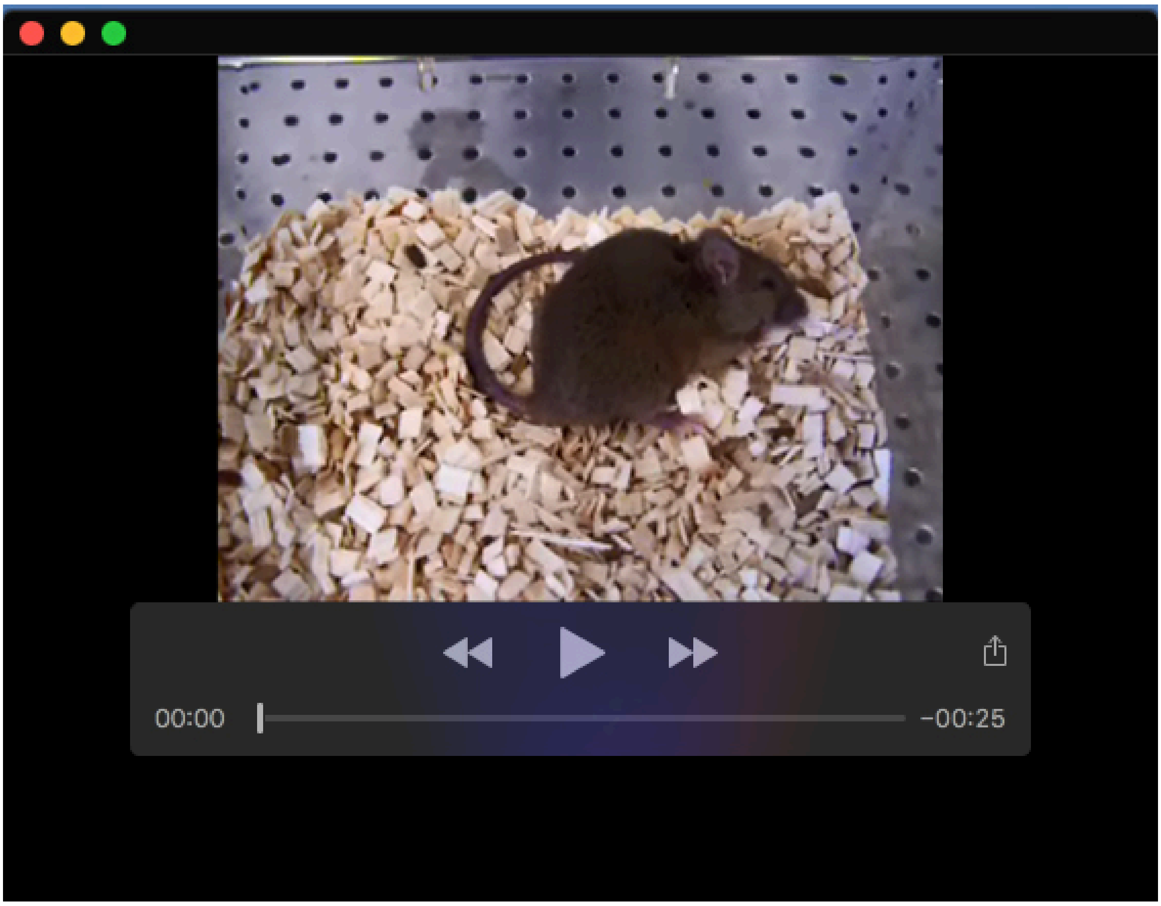

Movie 1. Movie depicting head tossing behaviour characteristic of *Jag1*$^{Ndr/Ndr}$ mice, as well as moving with head tilted to one side.

Development • Supplementary information
