# Supplementary material for: Jag1 represses Notch activation in lateral supporting cells and inhibits an outer hair cell fate in the medial cochlea
Source: Development. 2024 Nov 5;151(21):dev202949. doi: 10.1242/dev.202949 (PMC11574350; doi:10.1242/dev.202949)
Supplement: Supplementary file 1 [file develop-151-202949_review_history.pdf]

Jag1 represses Notch activation in lateral supporting cells and inhibits an outer hair cell fate in the medial cochlea

Sandra de Haan, Agustin A. Corbat, Christopher R. Cederroth, Lisa G. Autrum, Simona Hankeova, Elizabeth C. Driver, Barbara Canlon, Matthew W. Kelley and Emma R. Andersson
DOI: 10.1242/dev.202949

Editor: François Guillemot

Review timeline

Submission to Review Commons:	5 February 2024
Submission to Development	11 April 2024
Editorial decision:	15 April 2024
First revision received:	19 September 2024
Accepted:	25 September 2024

Reviewer 1

Evidence, reproducibility and clarity

Notch signaling regulates inner and middle ear morphogenesis and establishes a strict pattern of sensory cells in the organ of Corti in the mammalian cochlea. In the paper, the authors investigate the function of Jag1-mediated Notch activation in cochlear patterning and signaling using a novel Jag1 "Nodder" (Jag1Ndr/Ndr) mouse model of Alagille syndrome. In the transgenic mouse model, they found that the mice exhibited severe vestibular and auditory defects including an increase in ectopic inner hair cells, and a reduction in outer hair cells. By single-cell RNA study of the organ of Corti, the authors demonstrated a global dysregulation of genes associated with inner ear development and deafness. They observed that the role of Tbx2 in IHC specification is likely influenced by Notch signaling. This was a well-designed study with quality data that provided valuable information on the effect of dysregulated Jag1 on human Alagille syndrome. Given the recent success of a gene therapy clinical trial for human genetic hearing loss, this study helps to answer some key questions related to hearing, which should be of significance guiding future development of potential therapy.

To study how Jag1 insufficiency affects the development, the authors included the JagNdr/Ndr mouse model. To fully understand the characteristics of the Nodder mouse model, it's necessary to include the direct age-dependent comparison of the Jag1 level (by qPCR/and or Western blot) between Jag1+/+ v.s. from JagNdr/Ndr in Figure 1 at some selected stages to correlate the Jag1 insufficiency with the "Nodder" model. A spatial expression comparison of Jag1 between Jag1+/+ v.s. from JagNdr/Ndr from different the main age groups should be included in SFigure 2, together with Notch target genes.

Developmentally hair cells develop from the base to the apex starting from the IHC to OHC. The observation of the changes in HC pattern indi cates the impact of Notch in timing and maturation status of HC differentiation. Likely by the time when OHCs are supposed to be developed, which is dictated by the suppression of IHC and the activation of OHC signals, due to the dysregulation of Jag1, the IHC signaling cannot be sufficiently suppressed, whereas the OHC signaling cannot be

© 2024. Published by The Company of Biologists under the terms of the Creative Commons Attribution License (https://creativecommons.org/licenses/by/4.0/).

sufficiently activated. This has a positional effect as further it is from the IHCs, more mature OHC can develop. Could the authors dig deeper into the scRNAseq data to see if they can isolate the profile of extra IHCs in the JagNdr/Ndr mouse, to see if they can detect the expression of some OHC genes albeit at much lower levels?

It is difficult to dissect the contribution of middle ear malformation and inner ear defects to hearing loss in Alagille syndrome with the current model. For the development of any therapy, the two main factors have to be analyzed separately. One option is to generate an inner ear-specific JagNdr/Ndr model to bypass the middle ear issue, which can be evaluated for potential therapy. This part should be discussed.

In Figure 1, the author mentioned the major defects found in the vestibular system. Is there any difference in the vestibular system at the cellular level? Some evidence will be informative.

Scale bar missing in Figure1b and Figure1h.

The picture quality for Figure 4b is low, especially for F-actin staining. Please enhance the intensity.

Please mention the scale bar presented μm in the figure legends for Figure 2; Figure 3; SFigure 6.

Fig. 1g, poor quality. The WT cochlea looks severely disorganized.

Significance

Notch signaling regulates inner and middle ear morphogenesis and establishes a strict pattern of sensory cells in the organ of Corti in the mammalian cochlea. In the paper, the authors investigate the function of Jag1-mediated Notch activation in cochlear patterning and signaling using a novel Jag1 "Nodder" (Jag1Ndr/Ndr) mouse model of Alagille syndrome. In the transgenic mouse model, they found that the mice exhibited severe vestibular and auditory defects including an increase in ectopic inner hair cells, and a reduction in outer hair cells. By single-cell RNA study of the organ of Corti, the authors demonstrated a global dysregulation of genes associated with inner ear development and deafness. They observed that the role of Tbx2 in IHC specification is likely influenced by Notch signaling. This was a well-designed study with quality data that provided valuable information on the effect of dysregulated Jag1 on human Alagille syndrome. Given the recent success of a gene therapy clinical trial for human genetic hearing loss, this study helps to answer some key questions related to hearing, which should be of significance guiding future development of potential therapy.

Reviewer 2

Evidence, reproducibility and clarity

Alagille's syndrome is a developmental disorder in which the vast majority of patients have heterozygote mutations in the gene for the Notch ligand Jagged1. Patients with Alagille's have developmental defects in the heart, liver, eye and ear. This manuscript describes a hypomorphic new allele of Jag1 (Ndr mutation), in which homozygotes animals survive, allowing analysis of the postnatal and adult inner ear. The authors show that Jag1Ndr/Ndr mutants demonstrate hearing loss, vestibular defects, and patterning defects in the cochlea. Similar to previous studies of Jag1 in the ear, the authors find a decrease of outer hair cells, an increase of inner hair cells, and misplaced outer hair cells in the inner hair cell region. The authors perform scRNA-seq and analyze transcriptional effects in different cell populations. Interestingly, the authors present data that shows that the Notch pathway is upregulated in supporting cells at postnatal day 5, suggesting Jag1 may be playing an inhibitory role during postnatal maturation. The authors also show intriguing expression data regarding the OHC-like cells in the OHC region. Overall the study is well-performed and provides new information regarding the hair cell and supporting cell patterning defects caused by a reduction of Jag1-Notch signaling. However, most of the histological analysis was performed during development, and thus the origin of the severe hearing loss is not completely clear.

© 2024. Published by The Company of Biologists under the terms of the Creative Commons Attribution License (https://creativecommons.org/licenses/by/4.0/).

Comments

Major:

Fig1g shows a very abnormal cross section through the cochlear duct. There are no clearly visible Deiters' cells. Is this the case? Loss of outer hair cell function should only increase thresholds about 40dB, and there are increased thresholds reported here of 60+, despite remaining outer hair cells. This could be accounted for by the conduction defects, but also, there may be defects in the adult ear not observed earlier. Is there any inner hair cell loss? Deiter cell loss? Are inner and outer hair cell stereocilia normal? These may account for the severe hearing loss.

From the UMAP plot in Fig 2b, it seems that the scRNA-seq data did not reveal any change in cell identities in the Jag1Ndr/Ndr ears. This result is not really discussed in the results or discussion-particularly why the OHC-like cells, extra IHCs, and absent Hensen's cells are not revealed in this analysis.

It is difficult to know which cells are extra (+1), including inner hair cells and inner phalangeal cells. Since scRNAseq did not reveal a different gene signature for these 'extra' cells, it is more appropriate to just count them all together.

What is the rationale for reporting differences in the p-value that are not significant at the adjusted p-value? Since these are whole genome analysis it is only appropriate to report significance by adjusted p-values.

One of the novel aspects of this study is the finding that Notch components are upregulated in the Jag1Ndr/Ndr mutants (although some of these results are not significant at the adjusted p value). Given the potential significance that these results would indicate (including c-inhibition), it would be important to confirm upregulation of key Notch components in situ using RNA-scope or immunohistochemistry.

Additionally, a previous report has suggested that JAG1 mediates cis-inhibition in the medial region of the cochlea. The data presented here do not show an upregulation of Notch signaling in the medial supporting cells, suggesting this is not the case. This should be discussed.

Pg 9 Discussion: The sentence: "The JAG1NDR missense mutant is expressed in vivo, and traffics normally, but does not bind or activate NOTCH1", is somewhat misleading because it suggests this allele has no function. Based on the milder ear phenotype to null alleles as well as survival suggests that this allele is hypomorphic. This should be clarified and discussed.

Minor:

Pg 5 third paragraph, "Differential gene expression analysis identified 40 up- and 42-downregulated genes in Jag1Ndr/Ndr versus Jag1+/+ IPhCs, with pathway dysregulation similar to the pseudobulk analyses (Fig3c, Supp.Table 5,6)"-should be 40 downregulated and 42 upregulated.

Similarly: Pg 6 second paragraph: Differential gene expression analysis identified 1 up and 42-downregulated genes in Jag1Ndr/Ndr DCs versus Jag1+/+ DCs-should be 1 down and 42 up

Significance

This study corroborates and extends previous studies of the role of JAG1 in the inner ear. The role of JAG1 in cochlear development is not well understood compared to other Notch ligands, because it is expressed in the supporting cells and not the hair cells. The single cell RNAseq analysis presented here sheds new light on how JAG1 may be functioning postnatally. In addition, because this is a novel allele of JAG1 in which the homozygotes survive, we can further understand how the phenotype may affect hearing and balance in Alagille syndrome patients. This study will be interesting to those who study developmental biology, Notch signaling and the inner ear.

© 2024. Published by The Company of Biologists under the terms of the Creative Commons Attribution License (https://creativecommons.org/licenses/by/4.0/).

Reviewer 3

Evidence, reproducibility and clarity

Summary:

Patients with Alagille syndrome have impaired Notch signaling (~94% with JAG1 mutations) resulting in sensorineural and conductive hearing loss. To gain a better understanding of the genesis of these functional defects, the authors conduct a detailed examination of a mouse model of Alagille syndrome, called Nodder mice (Jag1 Ndr/Ndr). Consistent with previously reported phenotypes for Jag1 mutant mice, the authors observed severe vestibular and auditory defects that were accompanied by semicircular canal abnormalities, and defects in the patterning of the auditory sensory epithelium (medial boundary defect causing duplication of inner hair cells and inner phalangeal cells and reduction in outer hair cells and lateral supporting cells). What makes this study stand out from previous studies is the elegant use of single cell RNA-sequencing technology. Their scRNA_seq data suggest that 1) Jag1 lowers Notch signaling in lateral supporting cells and 2) Jag1 regulates the gene expression of outer hair cells. Further marker analysis revealed ectopic outer hair cell-like cells in the medial compartment and pillar cell region of Jag1 Ndr/Ndr mice. Surprisingly the outer hair cell-like cells closest to inner hair cells expressed Tbx2. Previous studies have shown that ectopic activation of Tbx2 is sufficient to convert outer hair cells into inner hair cells, suggesting that Jag1 deficiency may render these outer hair cells insensitive to Tbx2.

Overall, this is a very well-designed and executed study. The main conclusions of the study are well supported by the presented data and both data and methods are presented in a clear and detailed manner. I have only few suggestions for improvement:

Major comment:

The mutant form of Jagged1 in Nodder mice is trafficked to the cell surface, and while this mutant form of Jagged1 is incapable of activating the Notch1 receptor it may interact with "new" proteins, gaining new functions. My recommendation to the authors is to determine whether similar defects occur in conditional Jag1 knockout mice (increased Notch signaling in lateral supporting cells and presence of ectopic outer-hair cell like cells). The ability to disrupt Jag1 function at different stages of development may also help to determine why Jag1 deficiency renders some outer hair cells insensitive to Tbx2. If this is not possible due to time constrains I would recommend a more in-depth discussion of the limitations of using Nodder mice.

Minor comment:

The authors should provide a more detailed description of the Nodder mice (the nature of the mutation and how it may effect Notch1 and Notch2 receptor activation) in the introduction.

Significance

Strength of the study: The establishment and in-depth characterization of a Jag1 homozygous mutant mouse model (Nodder mice) to study the effects of Alagille syndrome on the auditory and vestibular system. Another strength is the characterization of the cell-type specific effects of Jag1 mutations using single cell transcriptomics.

Limitation of the study: It is unclear if the missense mutation in Jag1 that is present in Nodder mice causes "only" a loss of function. It is possible that a mutant form of Jag1 protein gains new functions (see also major comment).

Advance: This study demonstrates a novel role for the Notch ligand Jag1 in repressing Notch activation in lateral supporting cells and uncovers an involvement for Jag1-activated Notch signaling in inner versus outer hair cell specification and positioning.

© 2024. Published by The Company of Biologists under the terms of the Creative Commons Attribution License (https://creativecommons.org/licenses/by/4.0/).

Audience: This study will be of interest for both clinical and basic scientist as it provides novel insights into how Alagille syndrome effects the auditory system and novel mechanistic insights into the complex and cell-type specific role Notch signaling.

My field of expertise is: inner ear/ cochlea development, Notch signaling, cell fate specification and differentiation of cochlear supporting cells.

Manuscript number: RC- 2024-02380
Corresponding author(s): Emma R Andersson

1. General Statements

We sincerely appreciate the thorough and positive review provided by all reviewers. Their comments have provided valuable suggestions to improve and enhance clarity of our study on the role of Jag1-mediated Notch signaling in cochlear development, and its implications for Alagille syndrome. Furthermore, their feedback has underscored the significance of our study in elucidating patterning and hearing deficits, and its relevance for therapeutic considerations.

2. Description of the planned revisions

Comment from BioRxiv

In addition to comments from appointed reviewers, Jaime García-Añoveros emailed us with a comment on our BioRxiv preprint. Professor García-Añoveros was interested in our finding that*Tbx2* is expressed in OHC-like cells (Fig5), because his lab has shown that Tbx2 is an inner hair cell determinant (García-Añoveros et al., 2022). Fig 5 shows quantifications of *Tbx2* RNAscope punctae in sections, showing that *Tbx2* is expressed in *Jag1*$^{Ndr/Ndr}$ outer hair cell-like cells, in the inner hair cell compartment, at similar levels to that expressed by the extra inner hair cells also present in *Jag1*$^{Ndr/Ndr}$ mice. He suggested we perform RNAscope for *Tbx2* on wholemount cochlear preparations, to confirm the Fig 5 data from cross sections. While we are confident of our quantifications, which were based on optical slice sections <1mm thick (243.46nm), we acknowledge that the data and conclusions are controversial. To address this comment, we plan to perform wholemount RNAscope analyses of *Tbx2* in OHC-like cells, and quantify RNA scope signal in different cell types, to orthogonally test the previously obtained results.

Reviewer comments

We have already implemented some of the reviewer suggestions, as detailed under point 3, and the list below is therefore discontinuously numbered.

Reviewer 1

Comments regarding quality of images: the picture quality for Figure 4b is low, especially for F-actin staining. Please enhance the intensity. (check image). Fig. 1g, poor quality. The WT cochlea looks severely disorganized. (replace image)

Response

Figure 4b and Fig1g images will be improved or replaced. We plan a more extensive analysis of the adult phenotype, to also address comment #1 from Reviewer 2 (described below in response to Reviewer 2, #1).

Reviewer 2

Fig1g shows a very abnormal cross section through the cochlear duct. There are no clearly visible Deiters' cells. Is this the case? Loss of outer hair cell function should only increase thresholds about 40dB, and there are increased thresholds reported here of 60+, despite remaining outer hair cells. This could be accounted for by the conduction defects, but also, there may be defects in the adult ear not observed earlier. Is there any inner hair cell loss? Deiter cell loss? Are inner and outer hair cell stereocilia normal? These may account for the severe hearing loss.

Response

To further characterize the adult cochlear phenotype, we will quantify the number of IHCs, OHCs and SCs with immunohistological staining of cryosections from adult *Jag1*$^{Ndr/Ndr}$ mice, and address in the Discussion section how this phenotype relates to the observed hearing loss. Additionally, we

© 2024. Published by The Company of Biologists under the terms of the Creative Commons Attribution License (https://creativecommons.org/licenses/by/4.0/).

plan to analyze ABR wave-I characteristics of existing recordings to further study auditory nerve fiber responses and IHC function.
We have added a discussion of the relative contribution of middle and inner ear defects to the overall hearing loss in the Discussion section (lines 385-399), to also address comment #3 from reviewer 1 (below in section 3 "revisions that have been already incorporated in transferred manuscript").

Reviewer 2
What is the rationale for reporting differences in the p-value that are not significant at the adjusted p-value? Since these are whole genome analysis it is only appropriate to report significance by adjusted p-values.
One of the novel aspects of this study is the finding that Notch components are upregulated in the Jag1Ndr/Ndr mutants (although some of these results are not significant at the adjusted p value). Given the potential significance that these results would indicate (including c-inhibition), it would be important to confirm upregulation of key Notch components in situ using RNA-scope or immunohistochemistry.

Response
We agree that multiple hypothesis testing should be corrected for (with adjusted p values), which we have done in all analyses. However, we considered it relevant to report enriched or depleted genes that reached a meaningful fold difference and p-value threshold, even though the adjusted p-value threshold was not met. Our hope was that this would provide transparency and allow for consideration of the different sample sizes (different abundance of specific cell types), allowing the reader to explore the data. For further transparency, a distinction in labelling of significant adj. p-values and p-values was previously made in the original manuscript.

We thank the reviewer for pointing out that the Notch target gene upregulation is an interesting and novel finding. We will perform RNAscope experiments to validate the upregulation of Notch components and target genes at P5, including *Jag1, Jag2, Hes5, Nrarp, Tns1* and *Cxcl12*. Quantification of the RNA scope signal will also provide an alternative approach to testing whether the enrichment/upregulation of Notch target genes is statistically significant.

Reviewer 1
Text and figure comments: Scale bar missing in Figure1b and Figure1h. Please mention the scale bar presented mm in the figure legends for Figure 2; Figure 3; SFigure 6.

Response
Scale bar information will be added to the specified figures.

3. Description of the revisions that have already been incorporated in the transferred manuscript

Reviewer 1
Developmentally hair cells develop from the base to the apex starting from the IHC to OHC. The observation of the changes in HC pattern indicates the impact of Notch in timing and maturation status of HC differentiation. Likely by the time when OHCs are supposed to be developed, which is dictated by the suppression of IHC and the activation of OHC signals, due to the dysregulation of Jag1, the IHC signaling cannot be sufficiently suppressed, whereas the OHC signaling cannot be sufficiently activated. This has a positional effect as further it is from the IHCs, more mature OHC can develop. Could the authors dig deeper into the scRNAseq data to see if they can isolate the profile of extra IHCs in the JagNdr/Ndr mouse, to see if they can detect the expression of some OHC genes albeit at much lower levels?

Response
There were no significant gene expression differences between *Jag1*$^{Ndr/Ndr}$ and *Jag1*$^{+/+}$ IHCs. As we expect the *Jag1*$^{Ndr/Ndr}$ IHC pool to contain similar numbers of de facto IHCs and ectopic IHCs, failure to detect any differences suggests that the ectopic IHCs are transcriptionally similar to de facto IHCs. To further address the ectopic IHC signature, we subsetted, renormalized and reclustered the *Jag1*$^{Ndr/Ndr}$ and *Jag1*$^{+/+}$ IHCs. No *Jag1*$^{Ndr/Ndr}$-specific clusters were identified in this analysis (new

© 2024. Published by The Company of Biologists under the terms of the Creative Commons Attribution License (https://creativecommons.org/licenses/by/4.0/).

Supplementary Fig 4c). In addition, we analysed the expression of IHC- and OHC-specific markers to assess the faithfulness of *Jag1*$^{Ndr/Ndr}$ IHCs and OHCs. As reported in our original manuscript, *Jag1*$^{Ndr/Ndr}$ OHCs expressed lower levels of OHC markers. However, *Jag1*$^{Ndr/Ndr}$ IHCs were indistinguishable from *Jag1*$^{+/+}$ IHCs (new Supplementary Fig 4b). These new analyses also address comment #2 by Reviewer 2 (see below).

As the reviewer pointed out that development of HCs occurs from base to apex, we have added a quantification of apex and base regions of the P5 phenotype to Sfig5 and described this data in the Results section (lines 230-231).

Reviewer 1
It is difficult to dissect the contribution of middle ear malformation and inner ear defects to hearing loss in Alagille syndrome with the current model. For the development of any therapy, the two main factors have to be analyzed separately. One option is to generate an inner ear-specific JagNdr/Ndr model to bypass the middle ear issue, which can be evaluated for potential therapy. This part should be discussed.

Response
We agree that the relative contribution of middle and inner ear defects to hearing loss in a Jag1-compromised setting cannot be assessed with *Jag1*$^{Ndr/Ndr}$ mice. Generation of an inner-ear specific Jag1 Nodder model to bypass middle ear defects and address the relative contribution of middle and inner ear defects, would be technically challenging/impossible since the Nodder mouse model carries a single missense mutation in Jag1 and must be carefully maintained on a mixed genetic background to fully recapitulate Alagille syndrome. However, previous elegant work from other groups has dissected the function of Jag1 in supporting cells and neural crest, and how defects in each of these systems contribute to hearing loss. We therefore now comprehensively discuss this work by others (lines 385-399).

Reviewer 1
In Figure 1, the author mentioned the major defects found in the vestibular system. Is there any difference in the vestibular system at the cellular level? Some evidence will be informative.

Jag1$^{Ndr/Ndr}$ mice completely lack the posterior semicircular canal, which explains the head nodding behavior observed in our model, since the posterior semicircular canal detects head-tilting towards the shoulders. We have no data on the hair cells located in the saccule or utricle. Since the paper focusses on patterning and hearing, rather than balance, we consider further analysis of the vestibular system at cellular level outside of the scope of our paper.

Reviewer 2
From the UMAP plot in Fig 2b, it seems that the scRNA-seq data did not reveal any change in cell identities in the Jag1Ndr/Ndr ears. This result is not really discussed in the results or discussion- particularly why the OHC-like cells, extra IHCs, and absent Hensen's cells are not revealed in this analysis.

Response
In our scRNAseq dataset we were unable to identify, with certainty, an OHC-like population. After subsetting HCs, we did observe an additional OHC population exclusive to homozygous animals. However, after RNAscope validation, this population might have arisen from contamination with PCs. IHCs were transcriptionally similar between wildtype and homozygous animals, and we were unable to identify the ectopic IHCs. We additionally reported fewer to almost absent HeCs in the homozygous dataset. This data has been shown in the Results section (Fig2b) and in Supplementary Table 8 (number of cells per cell type) and has been discussed in the Discussion section. To further address the lack of separation of IHCs and ectopic IHCs, and failure to identify OHC-like cells, we have added additional panels assessing IHCs and OHC gene expression to SFigure4. This also addressed comments #2 addressed by Reviewer 1 (see above).

Reviewer 2

© 2024. Published by The Company of Biologists under the terms of the Creative Commons Attribution License (https://creativecommons.org/licenses/by/4.0/).

It is difficult to know which cells are extra (+1), including inner hair cells. Since scRNAseq did not reveal a different gene signature for these 'extra' cells, it is more appropriate to just count them all together.

Response
We have merged the quantification of IHCs and +1 IHCs to total IHCs in Fig4c. Separate original quantification of IHCs and +1 IHCs is reported in SFigure5, since the data presented in this way reflect a doubling of the IHC row.

Reviewer 2
Additionally, a previous report has suggested that JAG1 mediates cis-inhibition in the medial region of the cochlea. The data presented here do not show an upregulation of Notch signaling in the medial supporting cells, suggesting this is not the case. This should be discussed.

Response
It is indeed interesting to note that, although with comparable sample size for medial and lateral populations, upregulation of Notch activation is restricted to lateral SCs, and not, despite previous indications (Basch et al., 2016), observed in medial SC populations. We have discussed the possibility for cis-inhibition to a greater extent in the Discussion section (lines 310-311).

Reviewer 2 and Reviewer 3
Pg 9 Discussion: The sentence: "The JAG1NDR missense mutant is expressed in vivo, and traffics normally, but does not bind or activate NOTCH1", is somewhat misleading because it suggests this allele has no function. Based on the milder ear phenotype to null alleles as well as survival suggests that this allele is hypomorphic. This should be clarified and discussed.

The authors should provide a more detailed description of the Nodder mice (the nature of the mutation and how it may effect Notch1 and Notch2 receptor activation) in the introduction.

Response
We now introduce the *Nodder* mouse model (Hansson et al., 2010) and signaling defects to a greater extent in the Introduction section (lines 66-68).

Reviewer 2
Pg 5 third paragraph, "Differential gene expression analysis identified 40 up- and 42-downregulated genes in Jag1Ndr/Ndr versus Jag1+/+ IPhCs, with pathway dysregulation similar to the pseudobulk analyses (Fig3c, Supp.Table 5,6)"-should be 40 downregulated and 42 upregulated. Similarly: Pg 6 second paragraph: Differential gene expression analysis identified 1 up and 42-downregulated genes in Jag1Ndr/Ndr DCs versus Jag1+/+ DCs-should be 1 down and 42 up.

Response
Thank you for catching our accidental inversion here. The text has been corrected accordingly.

4. Description of analyses that authors prefer not to carry out

Please include a point-by-point response explaining why some of the requested data or additional analyses <u>might not be necessary or cannot be provided within the scope of a revision</u>. This can be due to time or resource limitations or in case of disagreement about the necessity of such additional data given the scope of the study. Please leave empty if not applicable.

Reviewer 1
To study how Jag1 insufficiency affects the development, the authors included the JagNdr/Ndr mouse model. To fully understand the characteristics of the Nodder mouse model, it's necessary to include the direct age-dependent comparison of the Jag1 level (by qPCR/and or Western blot) between Jag1+/+ v.s. from JagNdr/Ndr in Figure 1 at some selected stages to correlate the Jag1 insufficiency with the "Nodder" model. A spatial expression comparison of Jag1 between Jag1+/+ v.s. from JagNdr/Ndr from different the main age groups should be included in SFigure 2, together with Notch target genes.

© 2024. Published by The Company of Biologists under the terms of the Creative Commons Attribution License (https://creativecommons.org/licenses/by/4.0/).

The JAG1 Nodder mutation results in a hypomorphic ligand that is unable to bind and activate the Notch1 receptor (Hansson et al., 2010). The ligand itself, however, is still expressed, and its protein expression can even be upregulated in vivo (Hansson et al., 2010). Therefore, performing quantitative expression analysis of JAG1 expression (by qPCR or immunohistochemistry) would not provide insights into the levels of JAG1 activity. Instead, we show that there is decreased Notch target gene expression at the prosensory domain stage, as a proxy for Notch activation levels (SFigure2. A more detailed introduction of the model is provided in the Introduction (lines66-68), to also address a comment from Reviewer 2, #7 and Reviewer 3 comment #2.

Reviewer 3
The mutant form of Jagged1 in Nodder mice is trafficked to the cell surface, and while this mutant form of Jagged1 is incapable of activating the Notch1 receptor it may interact with "new" proteins, gaining new functions. My recommendation to the authors is to determine whether similar defects occur in conditional Jag1 knockout mice (increased Notch signaling in lateral supporting cells and presence of ectopic outer-hair cell like cells). The ability to disrupt Jag1 function at different stages of development may also help to determine why Jag1 deficiency renders some outer hair cells insensitive to Tbx2. If this is not possible due to time constrains, I would recommend a more in-depth discussion of the limitations of using Nodder mice.

Jag1 conditional knockout at various stages, has not been reported to result in ectopic OHC-like cells (Brooker et al., 2006; Chrysostomou et al., 2020; Gilels et al., 2022). However, two other *Jag1* missense mutants display atypical hair cells in the IHC compartment, which could be the OHC-like cells we report here (Kiernan et al., 2001; Tsai et al., 2001). Taken together, these data would suggest that Jag1 loss of function in supporting cells is not sufficient to result in OHC-like cells, but that constitutive Jag1 insufficiency can drive OHC-like cell formation. We now cite these data and discuss possible interpretations, as suggested (lines 324-331).

References

Basch, M. L., Brown, R. M., Jen, H.-I., Semerci, F., Depreux, F., Edlund, R. K., Zhang, H., Norton, C. R., Gridley, T., Cole, S. E., Doetzlhofer, A., Maletic-Savatic, M., Segil, N., & Groves, A. K. (2016). Fine-tuning of Notch signaling sets the boundary of the organ of Corti and establishes sensory cell fates. *ELife*, *5*, 841–850. https://doi.org/10.7554/eLife.19921

Brooker, R., Hozumi, K., & Lewis, J. (2006). Notch ligands with contrasting functions: Jagged1 and Delta1 in the mouse inner ear. *Development*, *133*(7), 1277–1286. https://doi.org/10.1242/dev.02284

Chrysostomou, E., Zhou, L., Darcy, Y. L., Graves, K. A., Doetzlhofer, A., & Cox, B. C. (2020). The notch ligand jagged1 is required for the formation, maintenance, and survival of Hensen's cells in the mouse cochlea. *Journal of Neuroscience*, *40*(49). https://doi.org/10.1523/JNEUROSCI.1192-20.2020

García-Añoveros, J., Clancy, J. C., Foo, C. Z., García-Gómez, I., Zhou, Y., Homma, K., Cheatham, M. A., & Duggan, A. (2022). Tbx2 is a master regulator of inner versus outer hair cell differentiation. *Nature*, *605*(7909). https://doi.org/10.1038/s41586-022-04668-3

Gilels, F. A., Wang, J., Bullen, A., White, P. M., & Kiernan, A. E. (2022). Deletion of the Notch ligand Jagged1 during cochlear maturation leads to inner hair cell defects and hearing loss. *Cell Death and Disease*, *13*(11). https://doi.org/10.1038/s41419-022-05380-w

Hansson, E. M., Lanner, F., Das, D., Mutvei, A., Marklund, U., Ericson, J., Farnebo, F., Stumm, G., Stenmark, H., Andersson, E. R., & Lendahl, U. (2010). Control of Notch-ligand endocytosis by ligand-receptor interaction. *Journal of Cell Science*, *123*(Pt 17), 2931–2942. https://doi.org/10.1242/jcs.073239

Kiernan, A. E., Ahituv, N., Fuchs, H., Balling, R., Avraham, K. B., Steel, K. P., & Hrabé de Angelis, M. (2001). The Notch ligand Jagged1 is required for inner ear sensory development. *Proceedings of the National Academy of Sciences of the United States of America*, *98*(7), 3873–3878. https://doi.org/10.1073/pnas.071496998

Tsai, H., Hardisty, R. E., Rhodes, C., Kiernan, A. E., Roby, P., Tymowska-Lalanne, Z., Mburu, P., Rastan, S., Hunter, A. J., Brown, S. D. M., & Steel, K. P. (2001). The mouse

© 2024. Published by The Company of Biologists under the terms of the Creative Commons Attribution License (https://creativecommons.org/licenses/by/4.0/).

slalom mutant demonstrates a role for Jagged1 in neuroepithelial patterning in the organ of Corti. *Hum Mol Genet*, *10*(5), 507–512. https://doi.org/10.1093/hmg/10.5.507

Author response to reviewers' comments

We sincerely appreciate the thorough and positive review provided by all reviewers. Their comments have provided valuable suggestions to improve and enhance clarity of our study on the role of Jag1-mediated Notch signaling in cochlear development, and its implications for Alagille syndrome. Furthermore, their feedback has underscored the significance of our study in elucidating patterning and hearing deficits, and its relevance for therapeutic considerations.

Reviewer #1 (Evidence, reproducibility and clarity (Required)):

Notch signaling regulates inner and middle ear morphogenesis and establishes a strict pattern of sensory cells in the organ of Corti in the mammalian cochlea. In the paper, the authors investigate the function of Jag1-mediated Notch activation in cochlear patterning and signaling using a novel Jag1 "Nodder" (Jag1Ndr/Ndr) mouse model of Alagille syndrome. In the transgenic mouse model, they found that the mice exhibited severe vestibular and auditory defects including an increase in ectopic inner hair cells, and a reduction in outer hair cells. By single-cell RNA study of the organ of Corti, the authors demonstrated a global dysregulation of genes associated with inner ear development and deafness. They observed that the role of Tbx2 in IHC specification is likely influenced by Notch signaling. This was a well-designed study with quality data that provided valuable information on the effect of dysregulated Jag1 on human Alagille syndrome. Given the recent success of a gene therapy clinical trial for human genetic hearing loss, this study helps to answer some key questions related to hearing, which should be of significance guiding future development of potential therapy.

To study how Jag1 insufficiency affects the development, the authors included the JagNdr/Ndr mouse model. To fully understand the characteristics of the Nodder mouse model, it's necessary to include the direct age-dependent comparison of the Jag1 level (by qPCR/and or Western blot) between Jag1+/+ v.s. from JagNdr/Ndr in Figure 1 at some selected stages to correlate the Jag1 insufficiency with the "Nodder" model. A spatial expression comparison of Jag1 between Jag1+/+ v.s. from JagNdr/Ndr from different the main age groups should be included in SFigure 2, together with Notch target genes.

Response
The JAG1 Nodder mutation results in a hypomorphic ligand that is unable to bind and activate the Notch1 receptor (Hansson et al., 2010). The ligand itself, however, is still expressed, and its protein expression can even be upregulated in vivo (Hansson et al., 2010). Therefore, performing quantitative expression analysis of JAG1 expression (by qPCR or immunohistochemistry) would not provide insights into the levels of JAG1 activity. However, the Jag1 prosensory domain is reduced in Jag1Ndr/Ndr mice at E14.5 (Supp Fig 3A, B, and at this time - when Jag1 is known to mediate lateral induction and Notch activation - Jag1Ndr/Ndr mice exhibit reduced Notch signaling with reduced HeyL, Hey1, Hey2 and Hes1 levels (Supp Fig 3C-E), corroborating that the Jag1Ndr allele is hypomorphic.
A more detailed introduction of the model is provided in the Introduction (lines 81-83).

Developmentally hair cells develop from the base to the apex starting from the IHC to OHC. The observation of the changes in HC pattern indicates the impact of Notch in timing and maturation status of HC differentiation. Likely by the time when OHCs are supposed to be developed, which is dictated by the suppression of IHC and the activation of OHC signals, due to the dysregulation of Jag1, the IHC signaling cannot be sufficiently suppressed, whereas the OHC signaling cannot be sufficiently activated. This has a positional effect as further it is from the IHCs, more mature OHC can develop. Could the authors dig deeper into the scRNAseq data to see if they can isolate the profile of extra IHCs in the JagNdr/Ndr mouse, to see if they can detect the expression of some OHC genes albeit at much lower levels?

© 2024. Published by The Company of Biologists under the terms of the Creative Commons Attribution License (https://creativecommons.org/licenses/by/4.0/).

Response
There were no significant gene expression differences between *Jag1*$^{Ndr/Ndr}$ and *Jag1*$^{+/+}$ IHCs (Supp Fig 5, Supp Table 5). As we expect the *Jag1*$^{Ndr/Ndr}$ IHC pool to contain similar numbers of de facto IHCs and ectopic IHCs, failure to detect any differences suggests that the ectopic IHCs are transcriptionally similar to de facto IHCs. To further address the ectopic IHC signature, we subsetted, renormalized and reclustered the *Jag1*$^{Ndr/Ndr}$ and *Jag1*$^{+/+}$ IHCs. No *Jag1*$^{Ndr/Ndr}$-specific clusters were identified in this analysis (new Supplementary Fig 5A). In addition, we analysed the expression of IHC- and OHC-specific markers to assess the faithfulness of *Jag1*$^{Ndr/Ndr}$ IHCs and OHCs. As reported in our original manuscript, *Jag1*$^{Ndr/Ndr}$ OHCs expressed lower levels of OHC markers. However, *Jag1*$^{Ndr/Ndr}$ IHCs were indistinguishable from *Jag1*$^{+/+}$ IHCs (new Supplementary Fig 5b).

As the reviewer pointed out that development of HCs occurs from base to apex, we have added a quantification of apex and base regions of the P5 phenotype to Supp Fig 6E and described this data in the Results section (lines 277-278).

It is difficult to dissect the contribution of middle ear malformation and inner ear defects to hearing loss in Alagille syndrome with the current model. For the development of any therapy, the two main factors have to be analyzed separately. One option is to generate an inner ear-specific JagNdr/Ndr model to bypass the middle ear issue, which can be evaluated for potential therapy. This part should be discussed.

Response
We agree that the relative contribution of middle and inner ear defects to hearing loss in a Jag1-compromised setting cannot be assessed with *Jag1*$^{Ndr/Ndr}$ mice. Generation of an inner-ear specific Jag1 Nodder model to bypass middle ear defects and address the relative contribution of middle and inner ear defects, would be technically challenging/impossible since the Nodder mouse model carries a single missense mutation in Jag1 and must be carefully maintained on a mixed genetic background to fully recapitulate Alagille syndrome. However, previous elegant work from other groups has dissected the function of Jag1 in supporting cells and neural crest, and how defects in each of these systems contribute to hearing loss. We therefore now comprehensively discuss this work by others (lines 467-484).

In Figure 1, the author mentioned the major defects found in the vestibular system. Is there any difference in the vestibular system at the cellular level? Some evidence will be informative.

Response
Jag1$^{Ndr/Ndr}$ mice completely lack the posterior semicircular canal, which explains the head nodding behavior observed in our model, since the posterior semicircular canal detects head-tilting towards the shoulders. We have no data on the hair cells located in the saccule or utricle. Since the paper focusses on Organ of Corti patterning and hearing, rather than balance, we consider further analysis of the vestibular system at cellular level outside of the scope of this manuscript, but an interesting topic for future research.

Scale bar missing in Figure1b and Figure1h.

Response
Thank you for noting these omissions, which have been corrected now (in revised Fig 1, it is Fig 1b and Fig 1i).

The picture quality for Figure 4b is low, especially for F-actin staining. Please enhance the intensity.

Response
We apologize for the pixelated images. We re-exported the images and replaced the panels, which now have better resolution.

© 2024. Published by The Company of Biologists under the terms of the Creative Commons Attribution License (https://creativecommons.org/licenses/by/4.0/).

Please mention the scale bar presented mm in the figure legends for Figure 2; Figure 3; Supp Figure 6.

Response
Thank you for noting these omissions, which have been corrected now.

Fig. 1g, poor quality. The WT cochlea looks severely disorganized.

Response
We have collected new ears, performed new stainings, and acquired new images to better represent the adult wild type and *Jag1*$^{Ndr/Ndr}$ cochlea, presented in new Fig 1G, and quantified in Fig 1H.

Reviewer #1 (Significance (Required)):

Notch signaling regulates inner and middle ear morphogenesis and establishes a strict pattern of sensory cells in the organ of Corti in the mammalian cochlea. In the paper, the authors investigate the function of Jag1-mediated Notch activation in cochlear patterning and signaling using a novel Jag1 "Nodder" (Jag1Ndr/Ndr) mouse model of Alagille syndrome. In the transgenic mouse model, they found that the mice exhibited severe vestibular and auditory defects including an increase in ectopic inner hair cells, and a reduction in outer hair cells. By single-cell RNA study of the organ of Corti, the authors demonstrated a global dysregulation of genes associated with inner ear development and deafness. They observed that the role of Tbx2 in IHC specification is likely influenced by Notch signaling. This was a well-designed study with quality data that provided valuable information on the effect of dysregulated Jag1 on human Alagille syndrome. Given the recent success of a gene therapy clinical trial for human genetic hearing loss, this study helps to answer some key questions related to hearing, which should be of significance guiding future development of potential therapy.

Response
We thank the reviewer for the careful appraisal of our work, highlighting the relevance for therapy, and kind words.

Reviewer #2 (Evidence, reproducibility and clarity (Required)):

Alagille's syndrome is a developmental disorder in which the vast majority of patients have heterozygote mutations in the gene for the Notch ligand Jagged1. Patients with Alagille's have developmental defects in the heart, liver, eye and ear. This manuscript describes a hypomorphic new allele of Jag1 (Ndr mutation), in which homozygotes animals survive, allowing analysis of the postnatal and adult inner ear. The authors show that Jag1Ndr/Ndr mutants demonstrate hearing loss, vestibular defects, and patterning defects in the cochlea. Similar to previous studies of Jag1 in the ear, the authors find a decrease of outer hair cells, an increase of inner hair cells, and misplaced outer hair cells in the inner hair cell region. The authors perform scRNA-seq and analyze transcriptional effects in different cell populations. Interestingly, the authors present data that shows that the Notch pathway is upregulated in supporting cells at postnatal day 5, suggesting Jag1 may be playing an inhibitory role during postnatal maturation. The authors also show intriguing expression data regarding the OHC-like cells in the OHC region. Overall the study is well- performed and provides new information regarding the hair cell and supporting cell patterning defects caused by a reduction of Jag1-Notch signaling. However, most of the histological analysis was performed during development, and thus the origin of the severe hearing loss is not completely clear.

Response
We acknowledge that our study primarily addressed the developmental roles of Jag1 in the establishment of inner ear structure and patterning. Further investigations will be required to elucidate the specific functions of Jag1 within distinct cell types and their contributions to hearing loss in Alagille syndrome, a multifaceted process that exhibits significant variability

© 2024. Published by The Company of Biologists under the terms of the Creative Commons Attribution License (https://creativecommons.org/licenses/by/4.0/).

across patients. Nevertheless, our findings offer valuable insights into several mechanisms likely involved in the pathology of hearing loss.

Comments

Major:

Fig1g shows a very abnormal cross section through the cochlear duct. There are no clearly visible Deiters' cells. Is this the case? Loss of outer hair cell function should only increase thresholds about 40dB, and there are increased thresholds reported here of 60+, despite remaining outer hair cells. This could be accounted for by the conduction defects, but also, there may be defects in the adult ear not observed earlier. Is there any inner hair cell loss? Deiter cell loss? Are inner and outer hair cell stereocilia normal? These may account for the severe hearing loss.

Response
We collected new inner ears, performed new stainings, and acquired new images to better characterize the adult wild type and *Jag1*$^{Ndr/Ndr}$ cochlea, presented in new Fig 1G, and quantified in Fig 1H. There is indeed a concomitant DC loss, proportional to the OHC loss. The extra inner hair cells and inner phalangeal cells persist to adulthood (Fig 1g,h). We also analyzed ABR wave-I to assess auditory nerve fiber responses and IHC function, presented in new Supp Fig 1, demonstrating higher latencies across all frequencies in Jag1Ndr/Ndr mice. Our data suggest that OHC and DC loss, together with potential conduction defects together increase hearing thresholds in Jag1Ndr/Ndr mice.

From the UMAP plot in Fig 2b, it seems that the scRNA-seq data did not reveal any change in cell identities in the Jag1Ndr/Ndr ears. This result is not really discussed in the results or discussion- particularly why the OHC-like cells, extra IHCs, and absent Hensen's cells are not revealed in this analysis.

Response
The shifts in cell numbers are corroborated by the scRNA seq cell numbers, but since dissociation artefacts or cell fragility (which could be influenced by genotype) can affect cell recovery, we did not focus on the prevalence of cells in the scRNAseq data. In our scRNAseq dataset we were unable to identify, with certainty, an OHC-like population. After subsetting hair cells, we did observe an additional OHC population exclusive to homozygous animals (Supp Fig 5). However, after RNAscope validation, this population might have arisen from contamination with PCs (Supp Fig 7). IHCs were transcriptionally similar between wildtype and homozygous animals, and we were unable to identify the ectopic IHCs based on transcriptomes (Supp Fig 5, described on page 8). We additionally reported fewer to almost absent HeCs in the homozygous dataset (Described on page 6: ". However, 43 *Jag1*$^{+/+}$ and only 3 *Jag1*$^{Ndr/Ndr}$ HeCs were retrieved in the scRNA seq" (Fig 2B, Supp. Table 8, number of cells per cell type)) and has been discussed in the Discussion section.

It is difficult to know which cells are extra (+1), including inner hair cells and inner phalangeal cells. Since scRNAseq did not reveal a different gene signature for these 'extra' cells, it is more appropriate to just count them all together.

Response
We have merged the quantification of IHCs and +1 IHCs to total IHCs in Fig4c. Separate original quantification of IHCs and +1 IHCs is reported in Supp Figure 6a, since the data presented in this way reflect a doubling of the IHC row.

What is the rationale for reporting differences in the p-value that are not significant at the adjusted p-value? Since these are whole genome analysis it is only appropriate to report significance by adjusted p-values.

One of the novel aspects of this study is the finding that Notch components are upregulated in the Jag1Ndr/Ndr mutants (although some of these results are not significant at the adjusted p value). Given the potential significance that these results would indicate (including c-

© 2024. Published by The Company of Biologists under the terms of the Creative Commons Attribution License (https://creativecommons.org/licenses/by/4.0/).

inhibition), it would be important to confirm upregulation of key Notch components in situ using RNA-scope or immunohistochemistry.

Response
We agree that multiple hypothesis testing should be corrected for (with adjusted p values), which we have done in all analyses. However, we considered it relevant to report enriched or depleted genes that reached a meaningful fold difference and unadjusted p-value threshold, even though the adjusted p-value threshold was not met. Our hope was that this would provide transparency and allow for consideration of the different sample sizes (different abundance of specific cell types), allowing the reader to explore the data. For further transparency, a distinction in labelling of significant adj. p-values and p-values was explicitly made in the original manuscript.

We thank the reviewer for pointing out that the Notch target gene upregulation is an interesting and novel finding. To validate the scRNAseq data, we performed RNAscope experiments to validate the upregulation of Notch components and target genes at P5, including focused on the canonical notch target genes Nrarp and Hes5. The company released a new kit version, and the new protocol was harsh on our tissue, leading to inconclusive results for other probes we included in these experiments (*Tns1* and *Cxcl12* (not included)). Nrarp and Hes5 RNA scope, as well as for Jag2, yielded robust and clean signal, which we quantified and present in Fig 3J,K,L. These new data demonstrated a statistically significant upregulation of Notch target genes in Dieter's cells, and upregulation of Jag2 in outer hair cells.

Additionally, a previous report has suggested that JAG1 mediates cis-inhibition in the medial region of the cochlea. The data presented here do not show an upregulation of Notch signaling in the medial supporting cells, suggesting this is not the case. This should be discussed.

Response
It is indeed interesting to note that, although with comparable sample size for medial and lateral populations, upregulation of Notch activation is restricted to lateral SCs, and not, despite previous indications (Basch et al., 2016), observed in medial SC populations. We have now extended our discussion regarding medial cis- inhibition to a greater extent in the Discussion section (Page 11, lines 365-368):

"Previous work has shown that medial SCs can experience cis-inhibition (Basch et al., 2016). However, data presented here suggest JAG1 might cis-inhibit Notch signalling in DCs but does not provide evidence for Jag1 cis-inhibited Notch signalling in IPhCs."

Pg 9 Discussion: The sentence: "The JAG1NDR missense mutant is expressed in vivo, and traffics normally, but does not bind or activate NOTCH1", is somewhat misleading because it suggests this allele has no function. Based on the milder ear phenotype to null alleles as well as survival suggests that this allele is hypomorphic. This should be clarified and discussed.

Response
The $Jag1^{Ndr/Ndr}$ homozygous phenotype in a pure genetic background (C3H) is embryonic lethal at around E11.5 due to vascular defects and hemorrhaging, similar to published Jag1 mutants (Hansson et al JCS 2010). The Jag1DSL/DSL mice are reported to be embryonic lethal at E10 (Xue et al *Human Molecular Genetics*, 1999), but it is not straightforward to compare phenotypes across genetic backgrounds, and the exact timing of embryonic death in the two strains has not been systematically compared. The $Jag1^{Ndr/Ndr}$ mice described here are outbred to a carefully controlled C3H/C57bl6 genetic background to allow embryos to survive, be born, and recapitulate Alagille syndrome. It is thus not possible to conclude that the $Jag1^{Ndr/Ndr}$ mice have an overtly milder phenotype than null alleles. Further context is now provided on Page 12, in the Discussion:

"The $Jag1^{Ndr/Ndr}$ model is a *Jag1* insufficiency model (Andersson et al., 2018; Hansson et al., 2010). The $JAG1^{NDR}$ protein cannot bind or activate NOTCH1, but displays some residual binding and activation of NOTCH2, and can bind NOTCH3. However, an unknown neomorphic function for $JAG1^{NDR}$ cannot be excluded. While OHC-like cells have not previously explicitly been described in the inner hair cell compartment of *Jag1*-compromised mice, two other *Jag1* missense

© 2024. Published by The Company of Biologists under the terms of the Creative Commons Attribution License (https://creativecommons.org/licenses/by/4.0/).

mutant mice – Slalom (Slm, *Jag1*$^{+/Slm}$) and Headturner (Htu, *Jag1*$^{+/Htu}$) mice – display atypical hair cells in the IHC compartment with OHC stereocilia features. (Kiernan et al., 2001b; Tsai et al., 2001b). The presence of atypical OHC-like cells in the IHC compartment are thus likely a product of dysregulated Jag1 signaling, rather than a consequence of neomorphic Jag1 function in *Jag1*$^{Ndr/Ndr}$ mice. However, rather than being placed lateral to the bona fide IHCs, as the *Jag1*$^{Ndr/Ndr}$ OHC-like cells are, the Slm and Htu atypical hair cells are placed medial to the IHC row, demonstrating that the placement of the extra OHCs is likely sensitive to the nature of the *Jag1* perturbation."

Minor:

Pg 5 third paragraph, "Differential gene expression analysis identified 40 up- and 42-downregulated genes in Jag1Ndr/Ndr versus Jag1+/+ IPhCs, with pathway dysregulation similar to the pseudobulk analyses (Fig3c, Supp.Table 5,6)"-should be 40 downregulated and 42 upregulated.

Similarly: Pg 6 second paragraph: Differential gene expression analysis identified 1 up and 42-downregulated genes in Jag1Ndr/Ndr DCs versus Jag1+/+ DCs-should be 1 down and 42 up

Thank you for catching our accidental inversion here. The text has been corrected accordingly.

Reviewer #2 (Significance (Required)):

This study corroborates and extends previous studies of the role of JAG1 in the inner ear. The role of JAG1 in cochlear development is not well understood compared to other Notch ligands, because it is expressed in the supporting cells and not the hair cells. The single cell RNAseq analysis presented here sheds new light on how JAG1 may be functioning postnatally. In addition, because this is a novel allele of JAG1 in which the homozygotes survive, we can further understand how the phenotype may affect hearing and balance in Alagille syndrome patients. This study will be interesting to those who study developmental biology, Notch signaling and the inner ear.

Reviewer #3 (Evidence, reproducibility and clarity (Required)):

Summary:

Patients with Alagille syndrome have impaired Notch signaling (~94% with JAG1 mutations) resulting in sensorineural and conductive hearing loss. To gain a better understanding of the genesis of these functional defects, the authors conduct a detailed examination of a mouse model of Alagille syndrome, called Nodder mice (Jag1 Ndr/Ndr). Consistent with previously reported phenotypes for Jag1 mutant mice, the authors observed severe vestibular and auditory defects that were accompanied by semicircular canal abnormalities, and defects in the patterning of the auditory sensory epithelium (medial boundary defect causing duplication of inner hair cells and inner phalangeal cells and reduction in outer hair cells and lateral supporting cells). What makes this study stand out from previous studies is the elegant use of single cell RNA-sequencing technology. Their scRNA_seq data suggest that 1) Jag1 lowers Notch signaling in lateral supporting cells and 2) Jag1 regulates the gene expression of outer hair cells. Further marker analysis revealed ectopic outer hair cell-like cells in the medial compartment and pillar cell region of Jag1 Ndr/Ndr mice. Surprisingly the outer hair cell-like cells closest to inner hair cells expressed Tbx2. Previous studies have shown that ectopic activation of Tbx2 is sufficient to convert outer hair cells into inner hair cells, suggesting that Jag1 deficiency may render these outer hair cells insensitive to Tbx2.

Overall, this is a very well-designed and executed study. The main conclusions of the study are well supported by the presented data and both data and methods are presented in a clear and detailed manner. I have only few suggestions for improvement:

Major comment:

© 2024. Published by The Company of Biologists under the terms of the Creative Commons Attribution License (https://creativecommons.org/licenses/by/4.0/).

The mutant form of Jagged1 in Nodder mice is trafficked to the cell surface, and while this mutant form of Jagged1 is incapable of activating the Notch1 receptor it may interact with "new" proteins, gaining new functions. My recommendation to the authors is to determine whether similar defects occur in conditional Jag1 knockout mice (increased Notch signaling in lateral supporting cells and presence of ectopic outer- hair cell like cells). The ability to disrupt Jag1 function at different stages of development may also help to determine why Jag1 deficiency renders some outer hair cells insensitive to Tbx2. If this is not possible due to time constrains I would recommend a more in-depth discussion of the limitations of using Nodder mice.

Response
Jag1 conditional knockout at various stages, has not been reported to result in ectopic OHC-like cells (Brooker et al., 2006; Chrysostomou et al., 2020; Gilels et al., 2022). However, two other *Jag1* missense mutants display atypical hair cells in the IHC compartment, which could be the OHC-like cells we report here (Kiernan et al., 2001; Tsai et al., 2001). Taken together, these data would suggest that Jag1 loss of function in supporting cells is not sufficient to result in OHC-like cells, but that constitutive Jag1 insufficiency can drive OHC-like cell formation. We now cite these data and discuss possible interpretations, as suggested (Page 12):

The $Jag1^{Ndr/Ndr}$ model is a *Jag1* insufficiency model (Andersson et al., 2018; Hansson et al., 2010). The $JAG1^{NDR}$ protein cannot bind or activate NOTCH1, but displays some residual binding and activation of NOTCH2, and can bind NOTCH3. However, an unknown neomorphic function for $JAG1^{NDR}$ cannot be excluded. While OHC-like cells have not previously explicitly been described in the inner hair cell compartment of *Jag1*-compromised mice, two other *Jag1* missense mutant mice – Slalom (Slm, $Jag1^{+/Slm}$) and Headturner (Htu, $Jag1^{+/Htu}$) mice – display atypical hair cells in the IHC compartment with OHC stereocilia features. (Kiernan et al., 2001b; Tsai et al., 2001b). The presence of atypical OHC-like cells in the IHC compartment are thus likely a product of dysregulated Jag1 signaling, rather than a consequence of neomorphic Jag1 function in $Jag1^{Ndr/Ndr}$ mice. However, rather than being placed lateral to the bona fide IHCs, as the $Jag1^{Ndr/Ndr}$ OHC-like cells are, the Slm and Htu atypical hair cells are placed medial to the IHC row, demonstrating that the placement of the extra OHCs is likely sensitive to the nature of the *Jag1* perturbation."

Minor comment:

The authors should provide a more detailed description of the Nodder mice (the nature of the mutation and how it may effect Notch1 and Notch2 receptor activation) in the introduction.

We have added details to the Introduction as follows: "The JAG1NDR missense mutant is expressed in vivo, and traffics normally (Hansson et al., 2010), and does not bind or activate NOTCH1, but can somewhat activate NOTCH2, resulting in Jag1 insufficiency (Andersson et al., 2018; Hansson et al., 2010)."

Reviewer #3 (Significance (Required)):

Strength of the study: The establishment and in-depth characterization of a Jag1 homozygous mutant mouse model (Nodder mice) to study the effects of Alagille syndrome on the auditory and vestibular system. Another strength is the characterization of the cell-type specific effects of Jag1 mutations using single cell transcriptomics.

Limitation of the study: It is unclear if the missense mutation in Jag1 that is present in Nodder mice causes "only" a loss of function. It is possible that a mutant form of Jag1 protein gains new functions (see also major comment).

Advance: This study demonstrates a novel role for the Notch ligand Jag1 in repressing Notch activation in lateral supporting cells and uncovers an involvement for Jag1-activated Notch signaling in inner versus outer hair cell specification and positioning.

Audience: This study will be of interest for both clinical and basic scientist as it provides novel

© 2024. Published by The Company of Biologists under the terms of the Creative Commons Attribution License (https://creativecommons.org/licenses/by/4.0/).

insights into how Alagille syndrome effects the auditory system and novel mechanistic insights into the complex and cell-type specific role Notch signaling.

My field of expertise is: inner ear/ cochlea development, Notch signaling, cell fate specification and differentiation of cochlear supporting cells.

References

Basch, M. L., Brown, R. M., Jen, H.-I., Semerci, F., Depreux, F., Edlund, R. K., Zhang, H., Norton, C. R., Gridley, T., Cole, S. E., Doetzlhofer, A., Maletic-Savatic, M., Segil, N., & Groves, A. K. (2016). Fine-tuning of Notch signaling sets the boundary of the organ of Corti and establishes sensory cell fates. *ELife*, *5*, 841–850. https://doi.org/10.7554/eLife.19921
Brooker, R., Hozumi, K., & Lewis, J. (2006). Notch ligands with contrasting functions: Jagged1 and Delta1 in the mouse inner ear. *Development*, *133*(7), 1277–1286. https://doi.org/10.1242/dev.02284
Chrysostomou, E., Zhou, L., Darcy, Y. L., Graves, K. A., Doetzlhofer, A., & Cox, B. C. (2020). The notch ligand jagged1 is required for the formation, maintenance, and survival of Hensen's cells in the mouse cochlea. *Journal of Neuroscience*, *40*(49). https://doi.org/10.1523/JNEUROSCI.1192-20.2020
García-Añoveros, J., Clancy, J. C., Foo, C. Z., García-Gómez, I., Zhou, Y., Homma, K., Cheatham, M. A., & Duggan, A. (2022). Tbx2 is a master regulator of inner versus outer hair cell differentiation. *Nature*, *605*(7909). https://doi.org/10.1038/s41586-022-04668-3
Gilels, F. A., Wang, J., Bullen, A., White, P. M., & Kiernan, A. E. (2022). Deletion of the Notch ligand Jagged1 during cochlear maturation leads to inner hair cell defects and hearing loss. *Cell Death and Disease*, *13*(11). https://doi.org/10.1038/s41419-022-05380-w
Hansson, E. M., Lanner, F., Das, D., Mutvei, A., Marklund, U., Ericson, J., Farnebo, F., Stumm, G., Stenmark, H., Andersson, E. R., & Lendahl, U. (2010). Control of Notch-ligand endocytosis by ligand-receptor interaction. *Journal of Cell Science*, *123*(Pt 17), 2931–2942. https://doi.org/10.1242/jcs.073239
Kiernan, A. E., Ahituv, N., Fuchs, H., Balling, R., Avraham, K. B., Steel, K. P., & Hrabé de Angelis, M. (2001). The Notch ligand Jagged1 is required for inner ear sensory development. *Proceedings of the National Academy of Sciences of the United States of America*, *98*(7), 3873–3878. https://doi.org/10.1073/pnas.071496998
Tsai, H., Hardisty, R. E., Rhodes, C., Kiernan, A. E., Roby, P., Tymowska-Lalanne, Z., Mburu, P., Rastan, S., Hunter, A. J., Brown, S. D. M., & Steel, K. P. (2001). The mouse slalom mutant demonstrates a role for Jagged1 in neuroepithelial patterning in the organ of Corti. *Hum Mol Genet*, *10*(5), 507–512. https://doi.org/10.1093/hmg/10.5.507

Original submission

First decision letter

MS ID#: DEVELOP/2024/202949

MS TITLE: Jag1 represses Notch activation in lateral supporting cells and inhibits an outer hair cell fate in the medial cochlea

AUTHORS: Sandra de Haan, Agustin A. Corbat, Christopher R. Cederroth, Lisa G. Autrum, Simona Hankeova, Elizabeth C. Driver, Barbara Canlon, Matthew W. Kelley and Emma R. Andersson

Thank you very much for transferring your Review Commons manuscript to Development, and for providing the reports from Review Commons' referees and your revision plan.

© 2024. Published by The Company of Biologists under the terms of the Creative Commons Attribution License (https://creativecommons.org/licenses/by/4.0/).

I have read these documents and my decision is to invite you to further revise your manuscript according to your revision plan and upload your revised manuscript when it is ready. If possible, your revised paper will be re-reviewed by one or more of the original referees, and acceptance of your manuscript will depend on your addressing satisfactorily the reviewers' major concerns. Please also note that Development will normally permit only one round of major revision. If it would be helpful, you are welcome to contact us to discuss your revision in greater detail. Please send us a point-by-point response indicating your plans for addressing the referees' comments, and we will look over this and provide further guidance.

Please attend to all of the reviewers' comments and ensure that you clearly highlight all changes made in the revised manuscript. Please avoid using 'Tracked changes' in Word files as these are lost in PDF conversion. I should be grateful if you would also provide a point-by-point response detailing how you have dealt with the points raised by the reviewers in the 'Response to Reviewers' box. If you do not agree with any of their criticisms or suggestions please explain clearly why this is so.

<u>Second decision letter</u>

MS ID#: DEVELOP/2024/202949

MS TITLE: Jag1 represses Notch activation in lateral supporting cells and inhibits an outer hair cell fate in the medial cochlea

AUTHORS: Sandra de Haan, Agustin A. Corbat, Christopher R. Cederroth, Lisa G. Autrum, Simona Hankeova, Elizabeth C. Driver, Barbara Canlon, Matthew W. Kelley and Emma R. Andersson

Thank you for sending your manuscript to Development through Review Commons.

I am delighted to tell you that your manuscript has been accepted for publication in Development, pending our standard publication integrity checks.

© 2024. Published by The Company of Biologists under the terms of the Creative Commons Attribution License (https://creativecommons.org/licenses/by/4.0/).
